# Ascorbic acid induces MLC2v protein expression and promotes ventricular-like cardiomyocyte subtype in human induced pluripotent stem cells derived cardiomyocytes

**DOI:** 10.7150/thno.80801

**Published:** 2023-07-03

**Authors:** Yu Gao, Liping Su, Yuhua Wei, Shihua Tan, Zhenyu Hu, Zhonghao Tao, Jean-Paul Kovalik, Tuck Wah Soong, Jianyi Zhang, Jun Pu, Lei Ye

**Affiliations:** 1 National Heart Research Institute Singapore, National Heart Centre Singapore, Singapore.; 2 Department of Cardiology, Ren Ji Hospital, School of Medicine, Shanghai Jiao Tong University, Shanghai 200127, China.; 3 Department of Biomedical Engineering, The University of Alabama at Birmingham, Birmingham, AL, USA.; 4 Department of Physiology, National University of Singapore, Singapore.; 5 Cardiovascular Diseases Translational Research Programme, Yong Loo Lin School of Medicine, National University of Singapore.; 6 Department of Thoracic and Cardiovascular Surgery, Nanjing First Hospital, Nanjing Medical University, Nanjing, Jiangsu, China.; 7 Programme in Cardiovascular and Metabolic Disorders, Duke-NUS, Singapore.

**Keywords:** human induced pluripotent stem cells, cardiomyocyte, electrophysiology

## Abstract

**Introduction:** The potentially unlimited number of cardiomyocyte (CMs) derived from human induced pluripotent stem cells (hiPSCs) *in vitro* facilitates high throughput applications like cell transplantation for myocardial repair, disease modelling, and cardiotoxicity testing during drug development. Despite promising progress in these areas, a major disadvantage that limits the use of hiPSC derived CMs (hiPSC-CMs) is their immaturity.

**Methods**: Three hiPSC lines (PCBC-hiPSC, DP3-hiPSCs, and MLC2v-mEGFP hiPSC) were differentiated into CMs (PCBC-CMs, DP3-CMs, and MLC2v-CMs, respectively) with or without retinoic acid (RA). hiPSC-CMs were either maintained up to day 30 of contraction (D30C), or D60C, or purified using lactate acid and used for experiments. Purified hiPSC-CMs were cultured in basal maturation medium (BMM) or BMM supplemented with ascorbic acid (AA) for 14 days. The AA treated and non-treated hiPSC-CMs were characterized for sarcomeric proteins (MLC2v, TNNI3, and MYH7), ion channel proteins (Kir2.1, Nav1.5, Cav1.2, SERCA2a, and RyR), mitochondrial membrane potential, metabolomics, and action potential. Bobcat339, a selective and potent inhibitor of DNA demethylation, was used to determine whether AA promoted hiPSC-CM maturation through modulating DNA demethylation.

**Results:** AA significantly increased MLC2v expression in PCBC-CMs, DP3-CMs, MLC2v-CMs, and RA induced atrial-like PCBC-CMs. AA treatment significantly increased mitochondrial mass, membrane potential, and amino acid and fatty acid metabolism in PCBC-CMs. Patch clamp studies showed that AA treatment induced PCBC-CMs and DP3-CMs adaptation to a ventricular-like phenotype. Bobcat339 inhibited MLC2v protein expression in AA treated PCBC-CMs and DP3-CMs. DNA demethylation inhibition was also associated with reduced TET1 and TET2 protein expressions and reduced accumulation of the oxidative product, 5 hmC, in both PCBC-CMs and DP3-CMs, in the presence of AA.

**Conclusions:** Ascorbic acid induced MLC2v protein expression and promoted ventricular-like CM subtype in hiPSC-CMs. The effect of AA on hiPSC-CM was attenuated with inhibition of TET1/TET2 mediated DNA demethylation.

## Introduction

The discovery of induced pluripotent stem cells (iPSCs) ushered in a new era in obtaining pluripotent stem cells from adult somatic cells. Human hiPSCs, like embryonic stem cells (ESCs), have self-renewal ability to proliferate indefinitely and yet retain the potential to differentiate into cells of the three primary germ layers (endoderm, mesoderm, and ectoderm) including cardiomyocytes (CMs). Cells derived from hiPSCs have a nearly identical genetic profile compared to the cell donor, which makes them useful for not only regenerative medicine, but also disease modeling research and drug screening.

Large quantities of human CMs have been successfully generated from hiPSCs using various differentiation protocols 1-3 and are being tested as cell transfer therapy for regenerating cardiac tissue in animal models 4-9. hiPSCs derived CMs (hiPSC-CMs) hold promise to serve as *in vitro* disease models for inherited and acquired heart diseases as the differentiated cells from patient specific hiPSCs retain disease-related phenotypes. Thus, the hiPSCs can serve as an *in vitro* model of pathogenesis, which will provide an innovative way to explore the molecular mechanisms of diseases and identify durgs for treatment.

Despite progress in the advancement in culturing techniques, the yield of atrial/ventricular hiPSC derived CMs may be protocol and cell line dependent. Most freshly differentiated hPSC-CMs display an immature CM phenotype which is different from ventricular CMs (CM) in terms of morphology, metabolism and functionality 10, 11. Since hiPSC-CMs display fetal-like atrial electrophysiology, immature hiPSC-CMs may increase risks for arrhythmias when used for myocardial repair 10 and may be not suitable for drug development. Most heart disease drugs target ventricular cardiomyocytes. Thus, it is crucial to have disease-specific ventricular CMs for *in vitro* for drug screening.

Studies have shown a variety of methods, such as long-term culture 12, 13, tissue engineering 14, 15, mechanical or electrical stimulus^5, 36^, metabolism 16, 17 and growth hormones 18 to promote the transition from immature CM to mature CMs at different levels. The maturation of cardiomyocytes is a complex process with extensive changes in morphology, electrophysiology, and metabolism defined by a collective set of changes observed at a physiological and molecular level 10, 11.

Ascorbic acid (AA) has been shown to improve CM differentiation from pluripotent stem cells 19-22. Although AA has been included in several formula of hiPSC-CM media due to its anti-oxidant effect 23-26, none studies reported its maturation effect and mechanism on hiPSC-CMs. In the current study, we explored the feasibility of AA for inducing ventricular-like hiPSC-CMs. We found that AA induced the gene and protein expression of MLC2v and promoted ventricular-like CM subtype in hiPSC-CMs.

## Methods

### Culture and differentiation of hiPSCs

Three hiPSC lines were used in this study: two were PCBC-hiPSCs and DP3-hiPSCs 9, 27-29 which have high CM differentiation efficiency, and one was MLC2v-mEGFP hiPSC which was purchased from Coriell Institute, USA. The MLC2v-mEGFP hiPSC is a transgenic reporter hiPSC line with GFP-tagged MLC2v gene. All cell lines were cultured in a feeder-free system with a mTeSR (STEMCELL Technologies, Canada) and passaged every 4-5 days with ReleSR (Stem Cell Tech., Canada) or Versene (Thermo Fisher) 9.

The differentiation protocol of hiPSCs into cardiomyocytes was described by Lian et al. 30. Briefly, hiPSCs were dissociated into single cells and cultured in mTeSR media for 4-5 days until confluence. On day 0, hiPSCs were cultured in RPMI medium supplemented with 1 x B27 without insulin (B27-) and 10 μM CHIR99021 for 24 h. On day 1, differentiation medium was changed to RPMI/B27- for 48 h. On day 3, cells were cultured in RPMI/B27- supplemented with 4 μM IWP2 for 48 h. On day 5, differentiation medium was changed to RPMI/B27- for 48 h. On day 7 of differentiation and every 3 days thereafter, cell culture medium was changed to RPMI/B27 medium. The differentiated hiPSCs would start contracting between day 8-10 of differentiation.

For induction of atrial-like hiPSC-CMs, 1 μM retinoic acid (RA) was added into differentiation medium on days 3-7 31, 32. Then, the contractile atrial-like hiPSC-CMs would either be maintained up to day 60 of contraction (D60C) or would be purified and used for experiments.

### Purification of hiPSC-CMs

To exclude non-CMs, we dissociated hiPSC-CMs into single cells on D7C to undergo purification 9, 33. Briefly, hiPSC-CMs were cultured in purification medium (Table 1) for 6 days 33. On D14C, purified cells would be used for experiment.

### Maturation of hiPSC-CMs

Purified hiPSC-CM were cultured in basal maturation medium (BMM) (Table 2). 150 μM AA was added into BMM to promote hiPSC-CM maturation. Cell culture medium was changed evey 2 days for 14 days. In parallel, hiPSC-CMs, which were maintained in RMPI supplemented with 50 x dilution of B27 up to D30C or D60C, would be used as non-treated control cells. The outline of experimental workflow was shown in Figure S1.

### Fluorescence immunostaining

To visualize contractile protein expression in hiPSC-CM after treatment, hiPSC-CMs were fixed with 4% paraformaldehyde for 20 min at room temperature and were permeabilized using 0.1% Triton X-100 at 4 ^o^C for 10 min. The cells were incubated with Ultra V Block (Thermo Fisher, USA) for 7 min and were incubated with primary antibodies (Table 3) at 4^ o^C overnight: mouse anti-cardiac troponin T (cTnT), rabbit anti-troponin I isoform 3 (TNNI3), mouse anti-atrial isoform myosin light chain 2 (MLC2a), rabbit anti-ventricular isoform myosin light chain 2 (MLC2v), rabbit anti-myosin heavy chain 6 (MYH6), and mouse anti-myosin heavy chain 7 (MYH7). On the second day, secondary antibodies, donkey anti-mouse IgG conjugated with Alexa Fluor® 555 or donkey-anti-rabbit IgG conjugated with Alexa Fluor® 488, were incubated with cells for 1 h at room temperature. Then, cell nuclei were counter-stained with 4′,6-diamidino-2-phenylindole (DAPI). Stained cells were mounted and visualized using Olympus IX73 microscope and Cell Sens Standard software (Olympus, Japan).

### Flow cytometry

To quantify protein expression level in hiPSC-CM after AA treatment, hiPSC-CMs were fixed, permeabilized, immunostained, and analyzed by flow cytometry using BD Fortessa (BD Biosciences, USA). The hiPSC-CMs with adequate size and granularity were included in the statistical analysis for assessing control protein expressions of cTnT, TNNI3, MLC2a, MLC2v, MYH6, and MYH7 (Table 4) 9, 34, 35. Acquired data was analyzed with FlowJo Version 7.6.2 (Treestar Software, Ashland, OR, USA).

### Quantatitive RT-PCR

To determine gene expression level of hiPSC-CM after AA treatment, hiPSC-CMs were collected to quantify gene expression levels of of contractile proteins. The isolation of total RNA and cDNA synthesis were carried out as previously described 36, 37. The quantitative PCR thermal cycling program for 40 cycles was: 1 cycle of enzyme activation at 95 ^o^C for 2 min, denaturation at 95 ^o^C for 5 s, annealing at 60 ^o^C for 20 seconds 27. The primers are listed in Table 5.

### Western Blot analysis

To determine protein expression level of hiPSC-CM after AA treatment, total protein of hiPSC-CMs was isolated using PhosphoSafe™ Extraction Reagent (Merck, Germany) and protein concentration was determined using Bradford reagent (Bio-Rad Laboratories, USA) as per instruction 7, 8, 37. Western Blot analysis of protein samples were performed as described previously 7, 8, 37. Proteins were separated on SDS-polyacrylamide gel and were transferred onto nitrocellulose membranes. After blocking with 5% non-fat milk in Tris-buffered saline Tween-20 buffer (25 mM Tris, pH 7.5, 150 mM NaCl, and 0.1% Tween-20), the blots were incubated with primary and corresponding secondary antibodies as listed in Table 6. The binding of the specific antibody was detected using the SuperSignal Chemiluminescent Substrate kit (Pierce, USA) and visualized by ChemiDoc™ XRS+ System (Bio-Rad, USA). The protein expression level was normalized by GAPDH and expressed as percentage of GAPDH 7, 8, 37.

### Mitochondrial staining

To determine mitochondrial membrane potential, mitochondrial staining was performed as described previously 28, 29. Briefly, hiPSC-CMs were cultured in 12-well plates and stained with mitochondria staining buffer using a Mitochondria Staining Kit (CS0390-1KT, Sigma, USA) for 30 min. The cells were washed with DPBS and visualized using an Olympus IX73 microscope and Cell Sens Standard software (Olympus, Japan). Images were taken at the same exposure time (50 milliseconds). For quantification of fluorescence intensity, ImageJ software was used to convert each fluorescent image into an 8-bit grayscale image, and quantitatively detected the average fluorescence intensity of each cell 29.

### DNA dot blot analysis of 5-hydroxymethylcytosine (5-hmC)

To determine whether AA promoted MLC2v protein expression through DNA demethylation, Bobcat339, a selective and potent inhibitor of ten-eleven translocation (TET) methylcytosine dioxygenases, was used to inhibit DNA demethylation through inhibiting TET1 and TET2 activities. Bobcat339 was added 30 min before AA was added into cell culture medium. DNA dot blot analysis of 5-hmC, an oxidized product of TET1/TET2, was performed. DNA was isolated using QIAamp DNA Mini Kit (Cat.No. 51304) and was denatured in 0.2 M NaOH at 95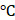 for 10 min. The solution was neutralized with 0.1 volume of 6.6 M ammonium acetate on ice for 1 min. DNA samples were spotted on Nitrocellulose membrane (Bio-Rad, USA), air-dried, washed in 2 x SSC buffer for 5 min, and air-dried at 80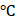 for 5min. After UV cross-linking for 5 min at 125 uJ / cm^2^, the membrane was blocked using 5% milk in TBST at room temperature for 1.5 h. Then, membrane was incubated with 1:2000 dilution of mouse anti-5-hmC antibody (MA5-23525, Invitrogen) in 5% milk in TBST at 4 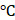 overnight. On the second day, the membrane was washed in 0.1% TBST buffer for 3 times and incubated in 5% milk TBST buffer containing 1:4000 dilution of goat anti-mouse IgG secondary antibody (NEF 822001 EA, Perkin Elmer) conjugated HRP for 1 h at room temperature. Then, membrane was washed in 0.1% TBST buffer for 4 times. The binding of the specific antibody was detected using the SuperSignal Chemiluminescent Substrate kit (Pierce, USA) and visualized by ChemiDoc™ XRS+ System (Bio-Rad, USA).

### Acylcarnitine profile measurement

To determine whether AA treatment will improve fatty acid oxidation-based metabolism, metabolomic study was performed as described previously 29. Briefly, hiPSC-CMs were washed with ice-cold PBS and were scraped off plates in ice-cold deionized water containing 0.6% formic acid. The solution was vortexed at 4^o^C to ensure cells were lysed. A sample of the lysate was set aside for protein determination. An equal volume of 100% acetonitrile was then added to the lysate. The lysates were then processed for determination of acyl-carnitine profile as previously described 38.

### Electrophysiology study

To determine electrophysiological properties, D30C or AA treated PCBC-CMs and DP3-CMs had electrophysiology studies to determine atrial-like or ventricular-like hiPSC-CMs phenotype as described 39. Briefly, whole-cell recording of action potentials was performed in spontaneously beating hiPSC-CMs using the current-clamp mode with an Axopatch 700B amplifier (Molecular Devices). The extracellular solution contained (in mM): NaCl 137, KCl 5.4, MgCl_2_ 1, NaH_2_PO_4_ 0.33, HEPES 10, glucose 10, CaCl_2_ 1.8, and pH adjusted to 7.4 with NaOH. The pipette (3-5 MΩ) solution contained (in mM): potassium gluconate 123, NaCl 9, MgCl_2_ 1.8, EGTA 0.9, HEPES 9, phosphocreatine 14, MgATP 4, and pH adjusted to 7.2 with KOH. The action potential durations after 80% repolarization (APD_80_) were analysed using pClamp 10 software (Molecular Devices, USA).

### Statistical analysis

Statistical analysis was performed using SPSS (version 18.0). All data were presented as mean ± standard deviation (SD). Comparisons among groups were analyzed for significance via one-way analysis of variance (ANOVA) with the Tukey correction. The numbers of ventricular-like hiPSC-CM and atrial-like hiPSC-CM in the D30C and AA treated cells were calculated based on action potential and weighted. As the total number was greater than 40 and no group had expected number less than 5, Pearson's chi-squared test was used. P < 0.05 was considered as statistical significance.

## Results

Using PCBC-CMs as cell models, sarcomeric proteins (MLC2v, TNNI3, and MYH7) and ion channel proteins (Kir2.1, Nav1.5, Cav1.2, SERCA2a, and RyR), mitochondrial membrane potential and biogenesis, metabolomics, and action potential were assessed. To understand potential signaling pathways, Bobcat339, a selective and potent inhibitor of DNA demethylation, was used to determine whether AA promoted hiPSC-CM maturation through modulating DNA demethylation. To exclude the effect from non-CM, we used purified hiPSC-CMs in all experiments. The lactate acid treatment purified hiPSC-CM from 83.8% to 95.2% based on cTnT protein expression assessed by FACS Figure S2.

### AA promoted MLC2v gene and protein expression in PCBC-CMs

We first determined the dose dependent effect of AA on MLC2v gene expression. AA at 150 μM concentration induced the highest MLC2v gene expression level Figure S3. Thus, 150 μM AA concentration was used in all the experiments.

**Figure 1:**
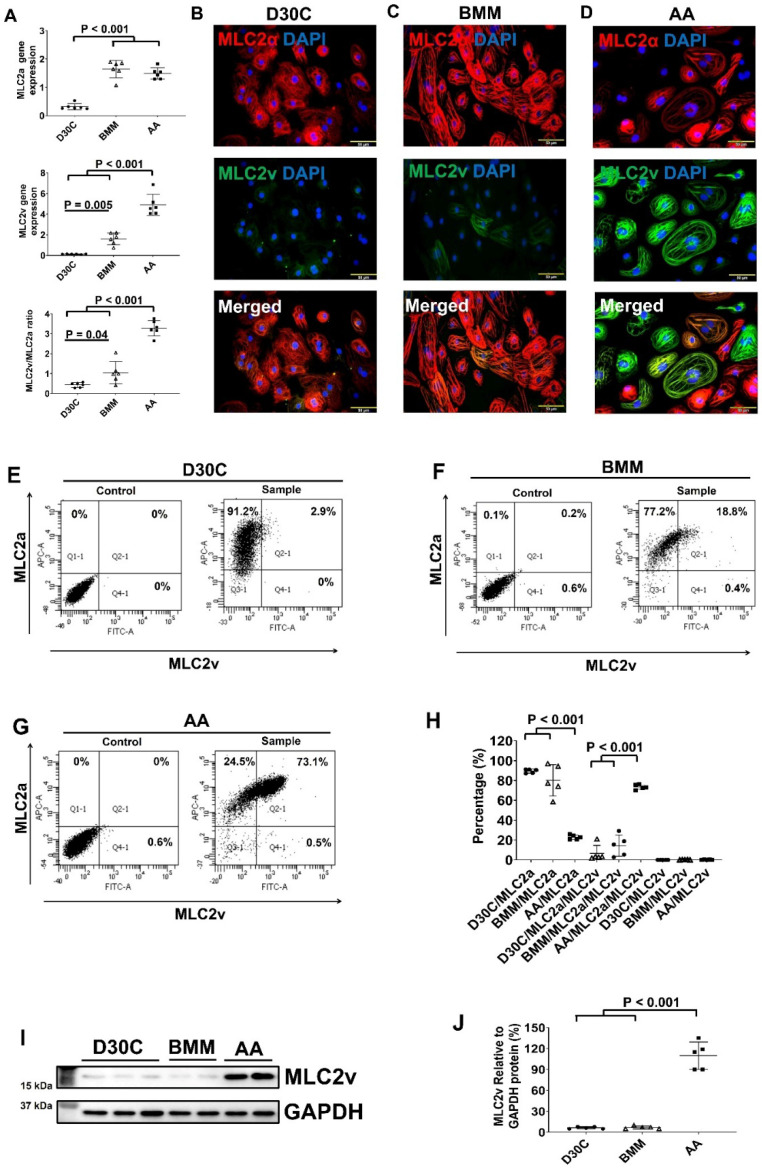
** Characterization of MLC2a and MLC2v gene and protein expression in PCBC-CMs. (A)** Gene expression levels of MLC2a and MLC2v in D30C, BMM, or AA treated PCBC-CMs. Fluorescence immunostaining of D30C **(B)**, BMM **(C)**, or AA treated **(D)** PCBC-CMs for MLC2a and MLC2v protein expressions. Representative flow cytometry results of D30C **(E)**, BMM **(F)**, or AA treated **(G)** PCBC-CMs for MLC2a, MLC2a/MLC2v, and MLC2v protein expressions and quantification **(H)**. Western Blot analysis of D30C, BMM, or AA treated PCBC-CMs for MLC2v protein expression **(I)** and quantification **(J)**. (n = 6 each for panel A and n = 5 each for panels H & J. Values are presented as the means ± SD. One-way ANOVA). (Bar = 50 μm).

Both BMM and AA treatments significantly increased MLC2a and MLC2v gene expression levels in PCBC-CMs as compared with D30C cells (Figure 1A). However, only AA induced the highest MLC2v gene expression level which was significantly higher than BMM treated cells. This resulted in the largest MLC2v : MLC2a ratio (3.28 ± 0.39), which is significantly higher than either D30C (0.43 ± 0.12, p < 0.001) or BMM treated (1.05 ± 0.55, p < 0.001) PCBC-CMs. Fluorescence immunostaining, FACS analysis, and Western Blot confirmed that AA promoted abundant MLC2v protein expression in PCBC-CMs (Figure 1B-J). The percentage of PCBC-CMs expressing only MLC2a significantly reduced, while cells expressing both MLC2a and MLC2V significantly increased and were higher than those of D30C and BMM treated cells (Figure 1H, p < 0.001 for all).

### AA promoted TNNI3 protein expression in PCBC-CMs

**Figure 2:**
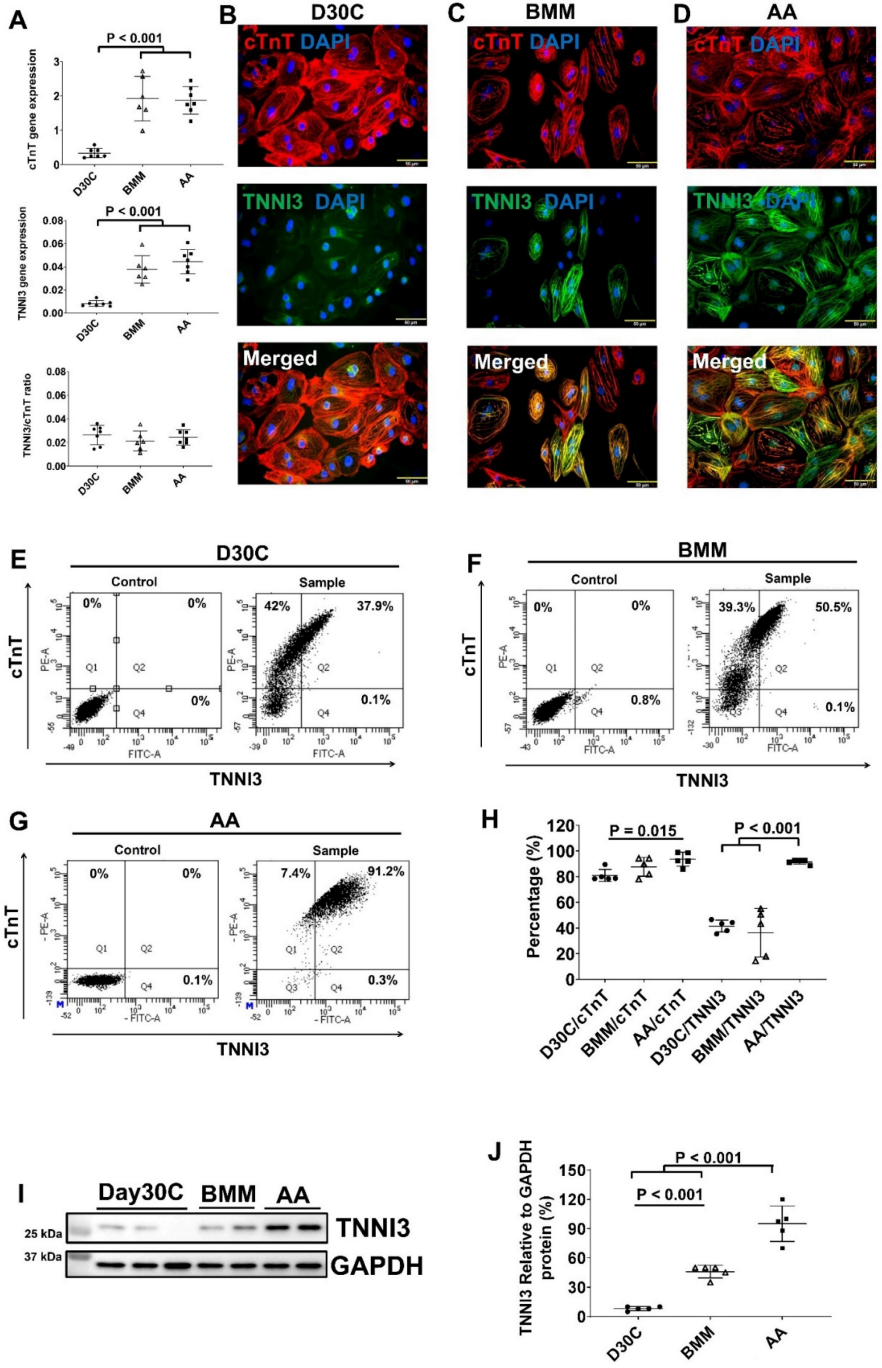
** Characterization of cTnT and TNNI3 gene and protein expression in PCBC-CMs. (A)** Gene expression levels of cTnT and TNNI3 in D30C, BMM, or AA treated PCBC-CMs. Fluorescence immunostaining of D30C **(B)**, BMM **(C)**, or AA treated **(D)** PCBC-CMs for cTnT and TNNI3 protein expressions. Representative flow cytometry images of D30C **(E)**, BMM **(F)**, or AA treated **(G)** PCBC-CMs for cTnT and TNNI3 protein expressions and quantification **(H)**. Western Blot analysis of D30C, BMM, or AA treated PCBC-CMs for TNNI3 protein expression **(I)** and quantification **(J)**. (n = 6 or 7 each for panel A and n = 5 each for panels H & J. Values are presented as the means ± SD. One-way ANOVA). (Bar = 50 μm).

Both BMM and AA significantly increased TNNI1 and TNNI3 gene expressions and no difference was observed between BMM and AA treatments (Figure 2A and Figure S4). The ratio of TNNI3:TNNI1 was the highest in the AA treated cells. Fluorescence immunostaining showed abundant TNNI3 protein expression in AA treated PCBC-CMs (Figure 2B-D). FACS analysis and Western Blot confirmed that AA promoted TNNI3 protein expression in CMs. About 91.4 ± 1.7% of PCBC-CMs expressed TNNI3 after AA treatment, which was significantly higher than D30C or BMM treated PCBC-CMs (p < 0.001 for both).

### AA promoted MHY7 gene and protein expression in PCBC-CMs

**Figure 3:**
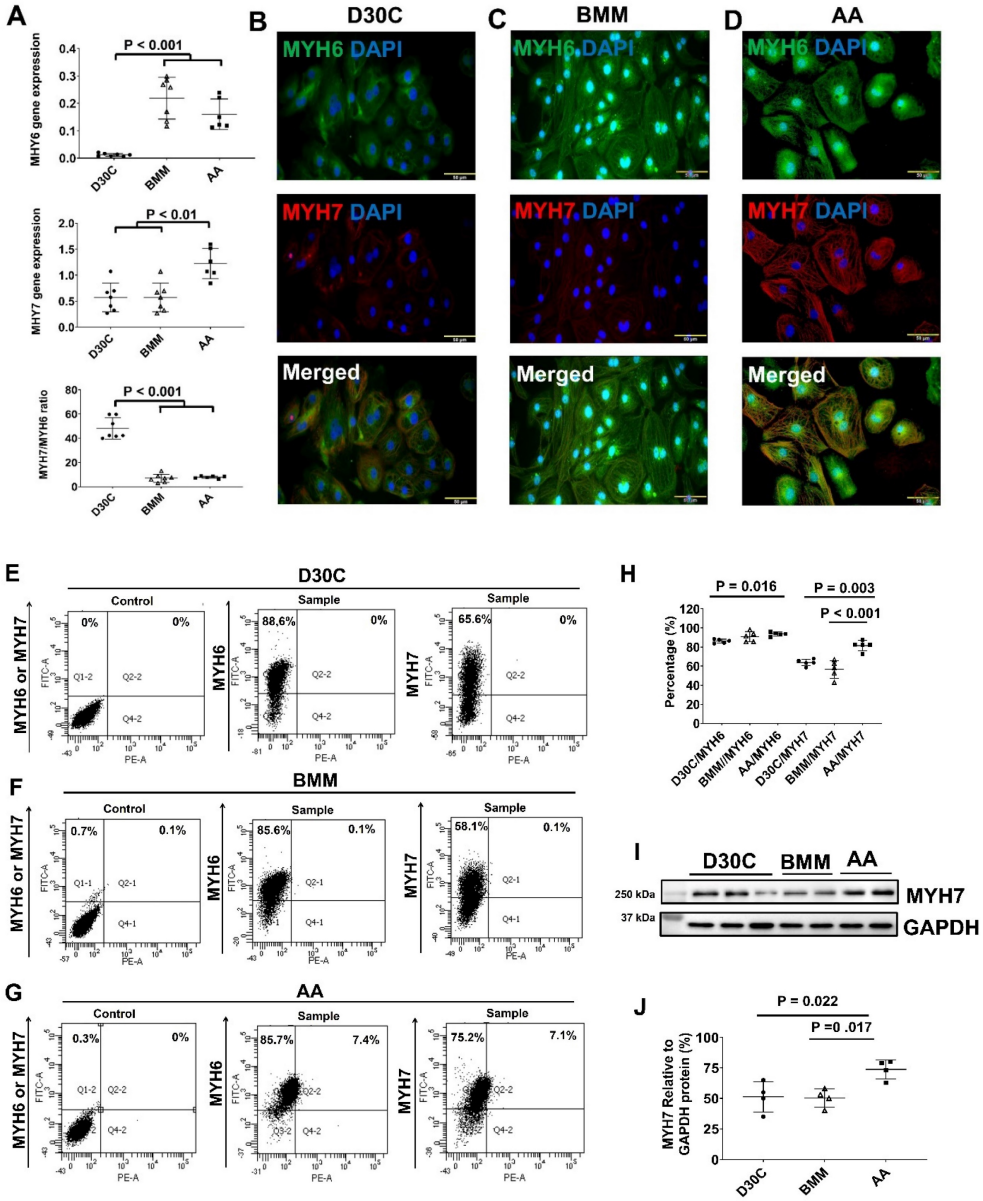

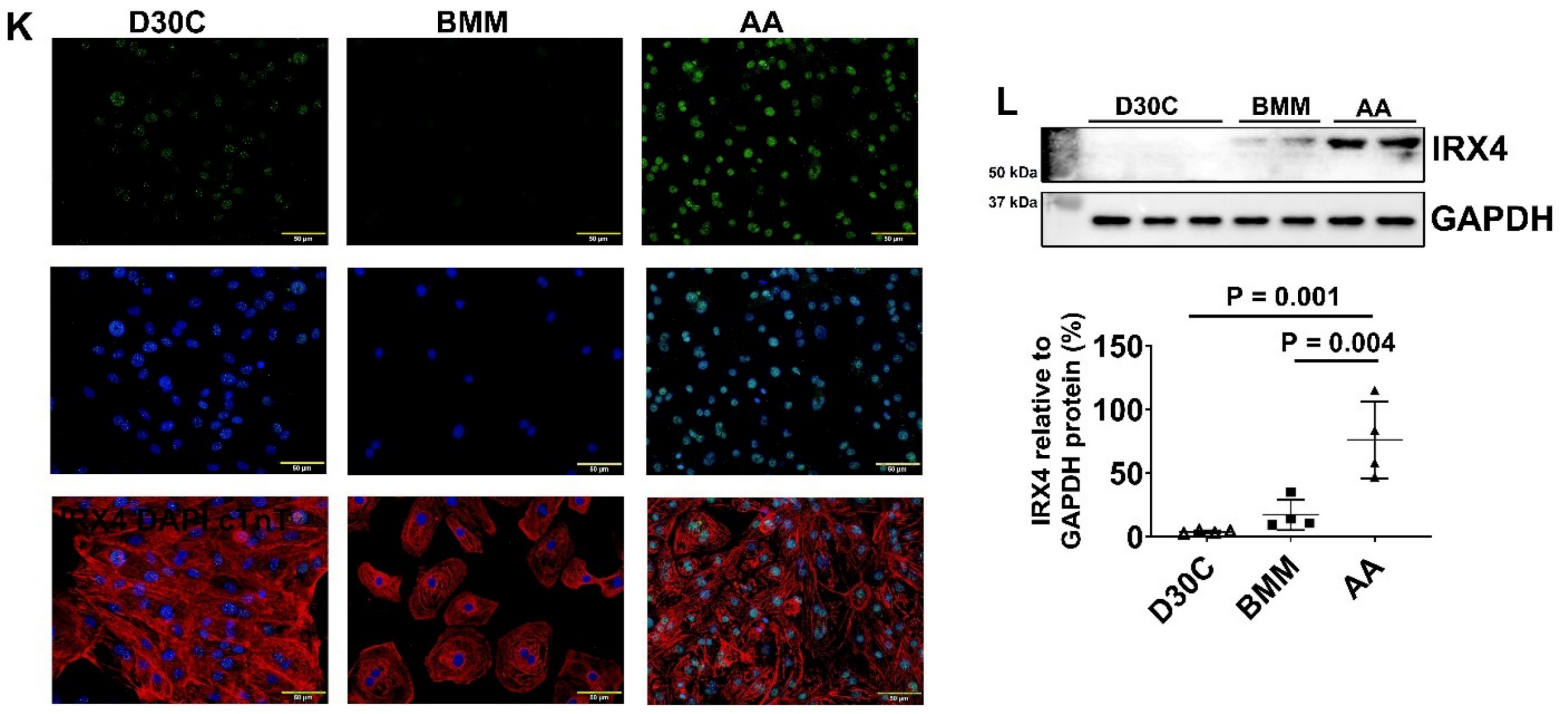
** Characterization of MYH6 and MYH7 gene and protein expressions in PCBC-CMs. (A)** Gene expression levels of MYH6 and MYH7 in D30C, BMM, or AA treated PCBC-CMs. Fluorescence immunostaining of D30C **(B)**, BMM **(C)**, or AA treated **(D)** PCBC-CMs for MYH6 and MYH7 protein expressions. Representative flow cytometry images of D30C **(E)**, BMM **(F)**, or AA treated **(G)** PCBC-CMs for MYH6 and MYH7 protein expressions and quantification **(H)**. Western Blot analysis of D30C, BMM, or AA treated PCBC-CMs for MYH7 protein expression **(I)** and quantification **(J)**. **(K)** Fluorescence immunostaining of D30C, BMM, or AA treated PCBC-CMs for IRX4 and cTnT protein expressions. **(L)** Western Blot of D30C, BMM, or AA treated PCBC-CMs for IRX4 protein expression. (n = 6 or 7 each for panel A, n = 5 each for panel H and n = 4 each for panels J and L). Values are presented as the means ± SD. One-way ANOVA). (Bar = 50 μm).

Both BMM and AA significantly increased MYH6 gene expression (Figure 3A). However, only AA significantly increased MYH7 gene expression as compared with D30C and BMM treated PCBC-CMs (Figure 3A). The ratio of MYH7:NYH6 was the highest in D30C cells (48.2 ± 8.8) which was significantly higher than BMM (7.0 ± 3.3) or AA (7.9 ± 1.0) treated PCBC-CMs (p < 0.001, for both). Fluorescence immunostaining, FACS analysis, and Western Blot confirmed that AA promoted abundant MYH7 protein expression in PCBC-CMs (Figure 3B-J). About 81.5 ± 5.4% of PCBC-CMs expressed MYH7 after AA treatment, which was significantly higher than those in D30C (p = 0.003) and BMM treated cells (p < 0.001).

### AA promoted IRX4 protein expression in PCBC-CMs

Fluorescence immunostaining and Western Blot showed that the protein expression of IRX4, a ventricular marker for CMs, was up-regulated in PCBC-CMs after AA treatment (Figure 3K) and Western Blot (Figure 3L). This indicates that AA promotes IRX4 expression.

### AA promoted RyR and Cav1.2 protein expression

**Figure 4:**
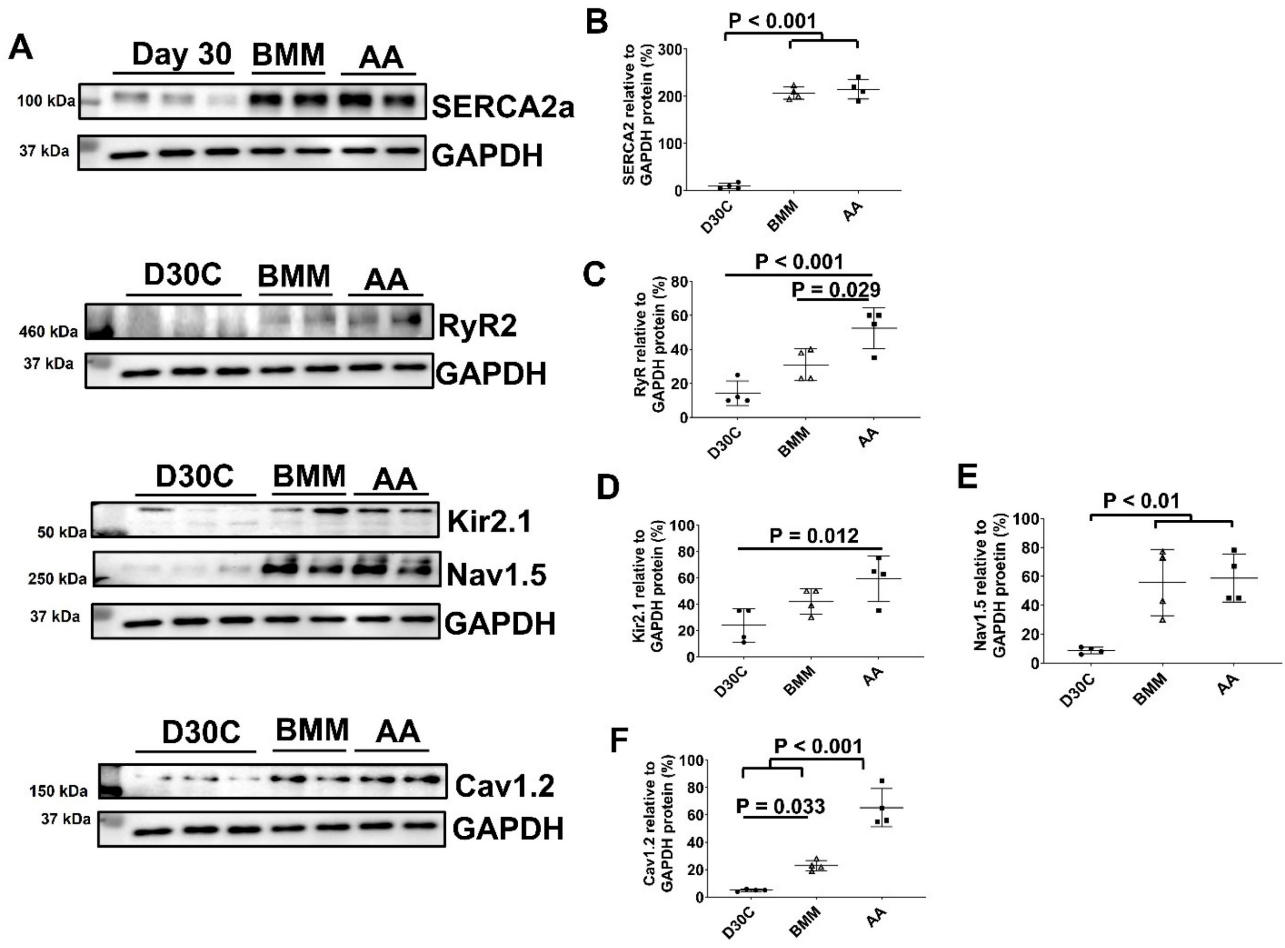
** Western Blot analysis for ion channel protein expression. (A)** Protein expression levels of SERCA2a, RyR2, Kir2.1, Nav1.5, and Cav1.2 in D30C, BMM, or AA treated PCBC-CMs. Quantification of SERCA2a **(B)**, RyR2 **(C)**, Kir2.1 **(D)**, Nav1.5 **(E)**, and Cav1.2 **(F)** protein expressions in D30C, BMM, or AA treated PCBC-CMs. (n = 4 each). Values are presented as the means ± SD. One-way ANOVA).

The ion channel protein expression levels of D30C, BMM, and AA treated PCBC-CMs were determined using Western Blot (Figure 4). Both BMM and AA treatments significantly increased SERCA2a, Nav1.5, and Cav1.2 protein expression levels as compared with D30C PCBC-CMs. AA treatment significantly increased RyR2 (p = 0.029) and Cav1.2 (p < 0.001) protein expression levels as compared with BMM treatment (Figure 4C & F).

### Both BMM and AA treatment increased mitochondrial membrane potential and mass in PCBC-CMs

**Figure 5:**
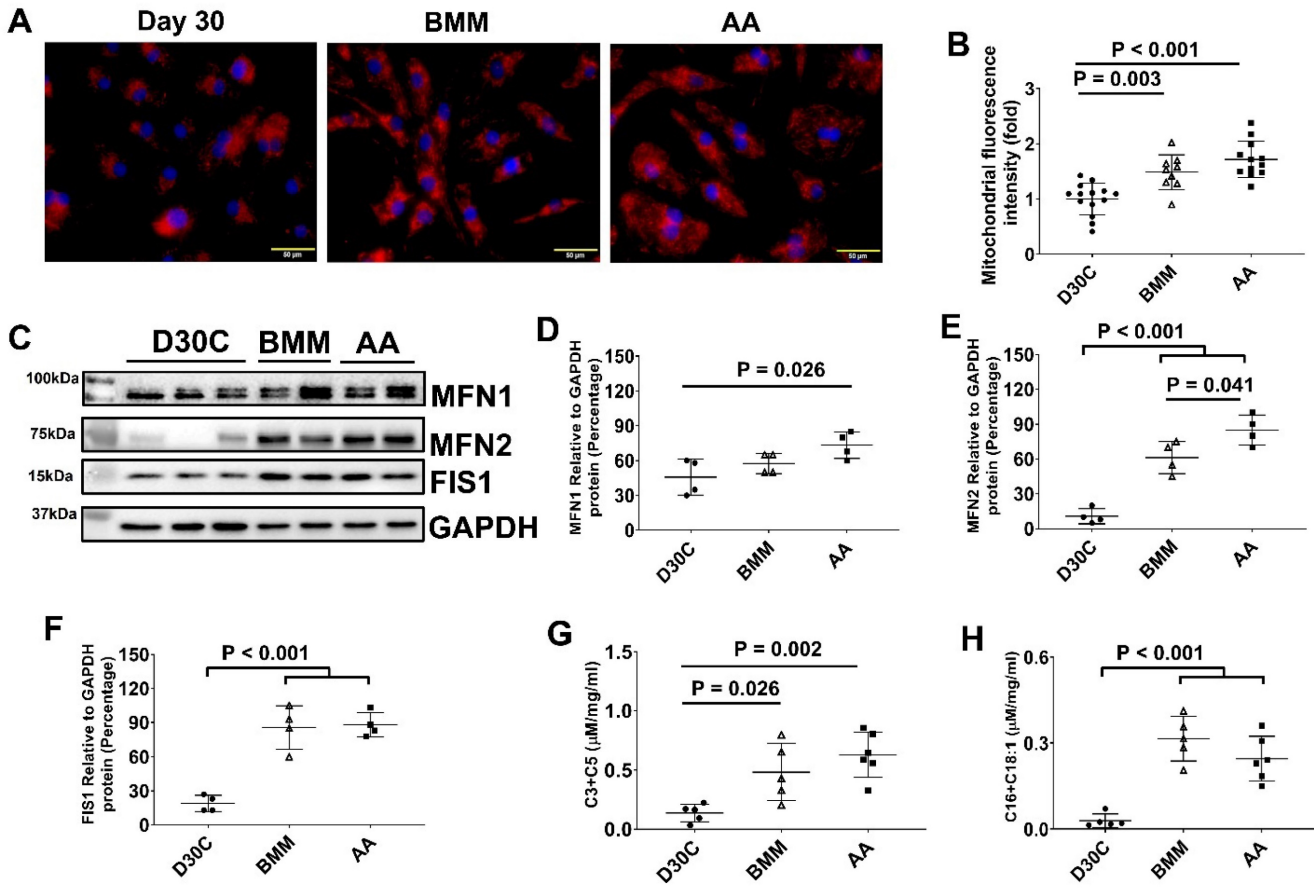
** Mitochondrial membrane potential and metabolism of PCBC-CMs. (A)** JC-1 staining to show mitochondrial membrane potential.** (B)** Quantification of fluorescence intensity based on JC-1 staining. **(C)** Western Blot analysis for protein expressions of MFN1, MFN2, and FIS1. Quantification of MFN1 **(D)**, MFN2 **(E)**, and FIS1 **(F)** protein expressions in D30C, BMM, or AA treated PCBC-CMs. Metabolism of C3 + C5 **(G)** and C16 + C18:1** (H)** in D30C, BMM or AA treated PCBC-CMs. (n = 9 - 14 for panel B, n = 4 each for panels D - F, n = 5 - 6 each for panels G & H. Values are presented as the means ± SD. One-way ANOVA). (Bar = 50 μm).

JC-1 staining of hiPSC-CMs showed that the majority of mitochondria located in the perinuclear region in D30C PCBC-CMs, while they were evenly distributed in the cytoplasm of BMM and AA treated PCBC-CMs (Figure 5A). Fluorescence intensity, an indicator of mitochondrial membrane potential and mass, of each CM was significantly high in both BMM and AA treated cells as compared with D30C cells (Figure 5B). Although there is a trend of higher fluorescence intensity in AA treated cells, no significant difference was observed between BMM and AA treated cells.

As mitochondrial biogenesis is determined by a dynamic equilibrium between organelle fusion and fission, the protein expression level of MFN1, MFN2, and FIS1 were determined using Western Blot. As shown in Figure 5C-F, both BMM and AA significantly increased MNF2 and FIS1 protein expression as compared with D30C cells. In addition, AA treatment resulted in further increased MFN2 protein expression as compared with BMM treated cells, suggesting that AA may promote mitochondrial biogenesis.

### Regardless of AA, BMM treatment increased amino acid and fatty acid metabolisms in PCBC-CMs

We measured acylcarnitines, which are representatives of mitochondrial fuel metabolism, in hiPSC-CMs. The C3 and C5 acylcarnitines, which are derived from catabolism of branched-chain amino acids, increased significantly in both BMM and AA treated cells. C16 and C18:1 acylcarnitines, which are derived from catabolism of long-chain fatty acid fuels, were increased in BMM and AA treated cells as compared with D30C cells (Figure 5G & H). The acylcarnitine results indicate that increased catabolism of amino acid and fatty acid fuels in both BMM and AA treated PCBC-CMs.

### AA promoted PCBC-CMs to adapt to ventricular-like CM electrophysiology profile

**Figure 6:**
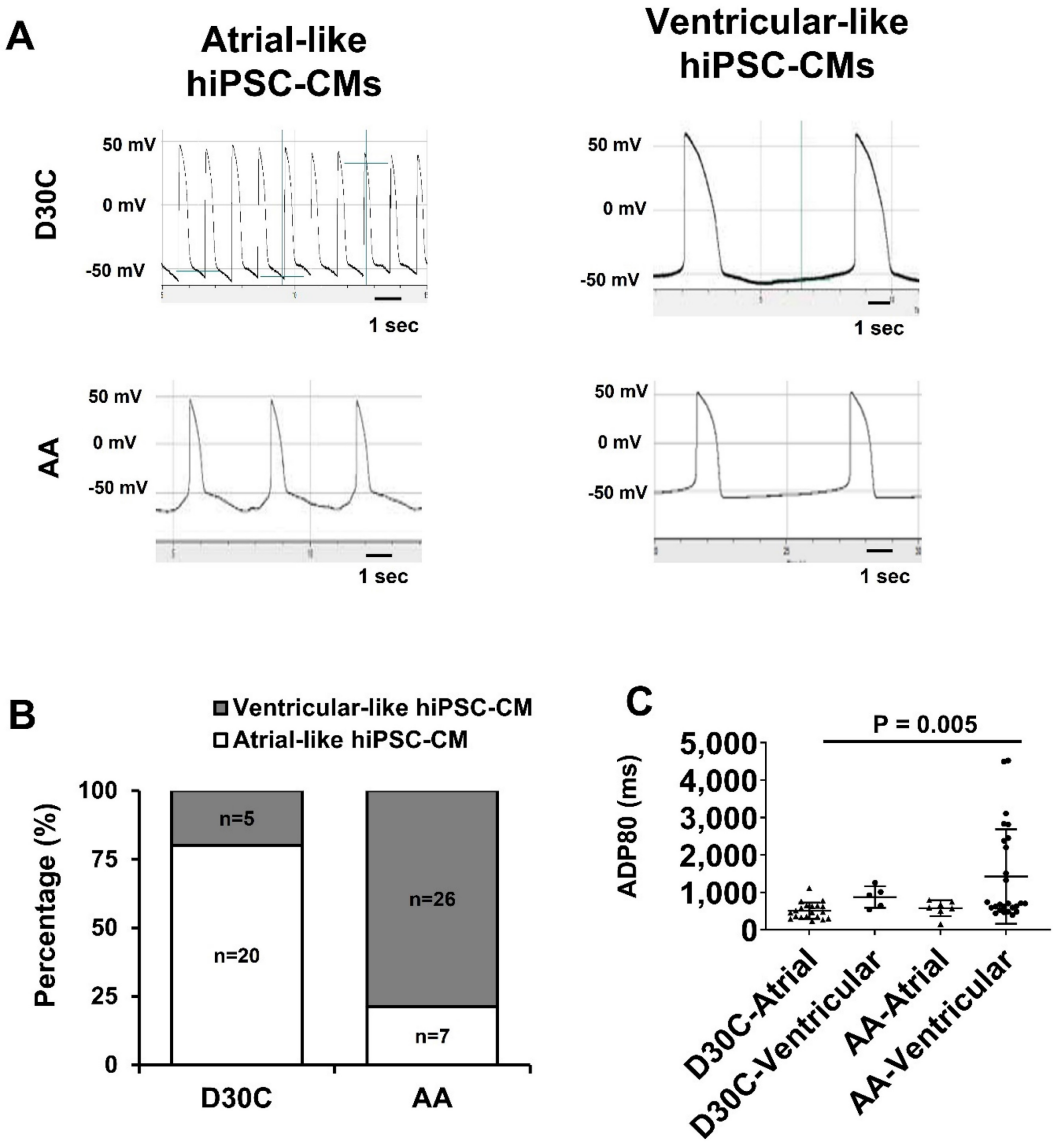
** Patch clamp analysis of D30C and AA treated PCBC-CMs**. **(A)** Action potential of atrial- or ventricular-like cells in D30C or AA treated PCBC-CMs. **(B)** Percentage of atrial or ventricular-like cells in D30C or AA treated PCBC-CMs based on action potential. **(C)** ADP80 of atrial or ventricular-like cells in D30C or AA treated PCBC-CMs. (n = 20 for D30C-Atrial, n = 5 for D30C-Ventricular, n = 7 for AA-Atrial, and n = 26 for AA-Ventricular). Values are presented as the means ± SD. Pearson's chi-squared test or One -way ANOVA).

Considering that AA prominently promoted MLC2v protein expression, patch clamp was used to determine whether AA treated PCBC-CMs would acquire ventricular CM action potential profile. Patch clamp studies demonstrated that 80% of D30C PCBC-CMs showed atrial-like action potential and only 20% of them showed ventricular-like action potential (Figure 6A & B). One the contrary, AA treatment significantly increased ventricular-like CM to 78.8% (p < 0.001), suggesting that AA promoted conversion of atrial-like CMs into ventricular-like CMs. Although the longest ADP80 was in AA treated ventricular-like-PCBC-CMs, it was only significantly longer than D30C atrial-like CMs.

### AA promoted MLC2v, TNNI3, MYH7, and IRX4 protein expressions in D60C PCBC-CMs

**Figure 7:**
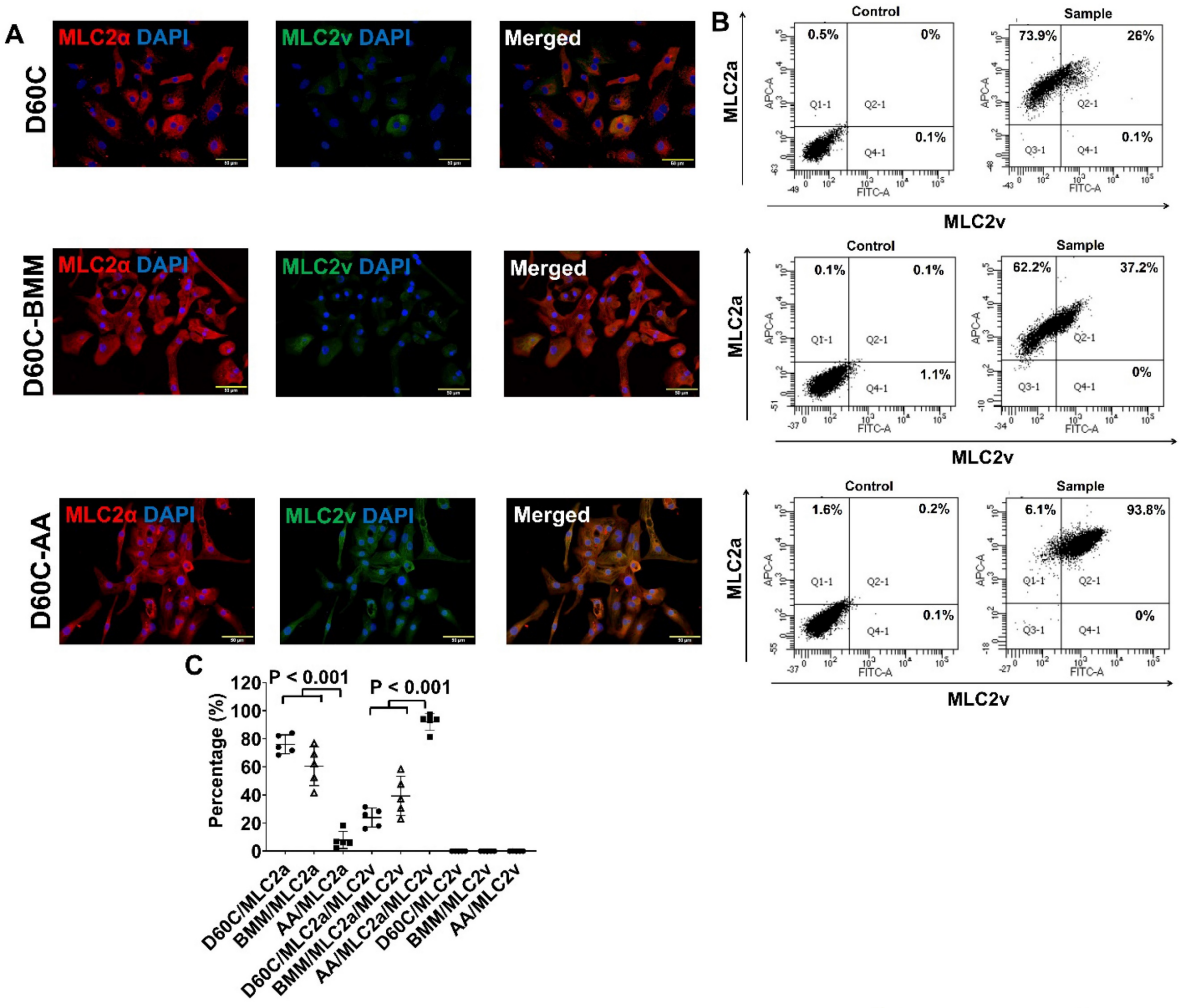

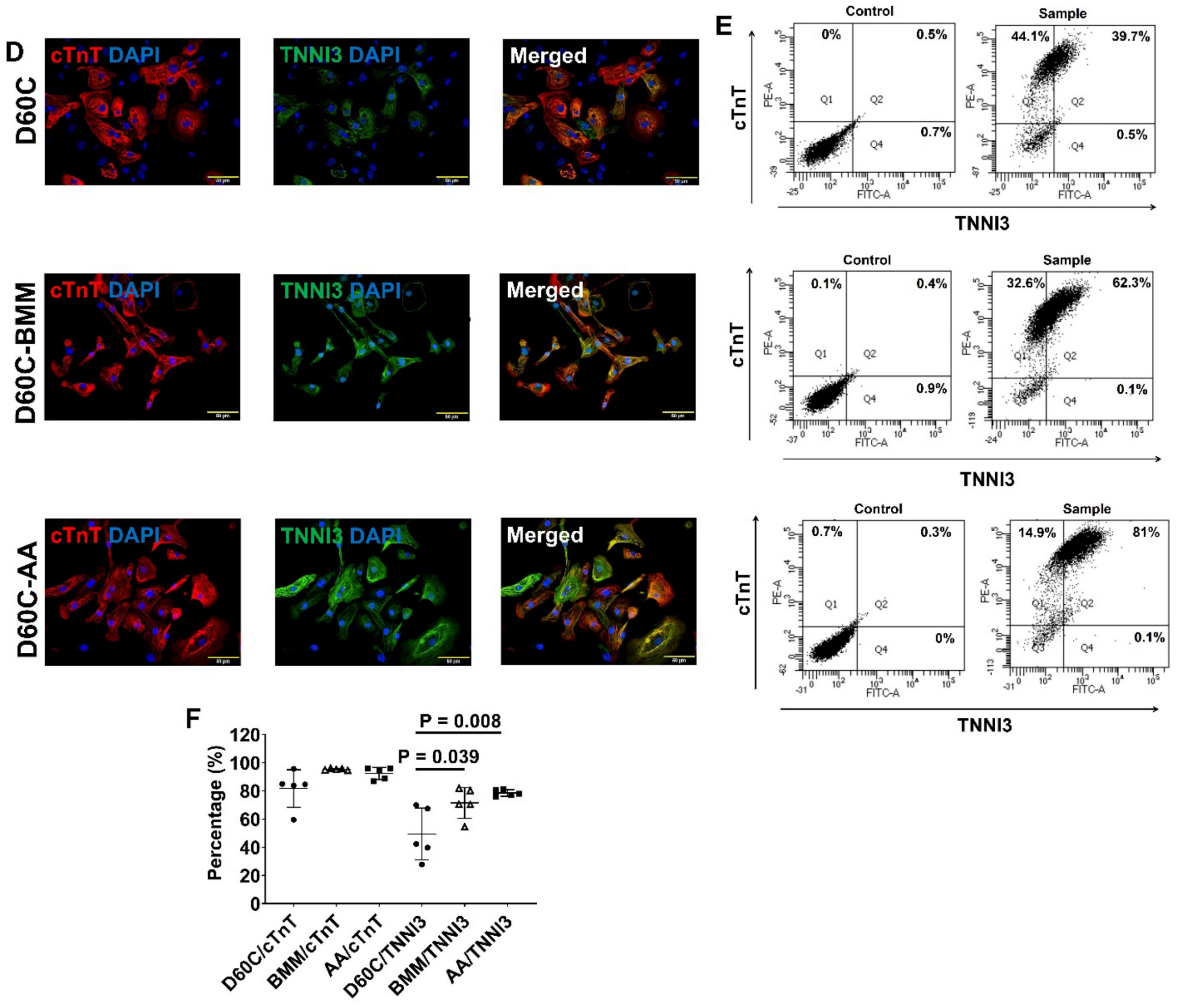

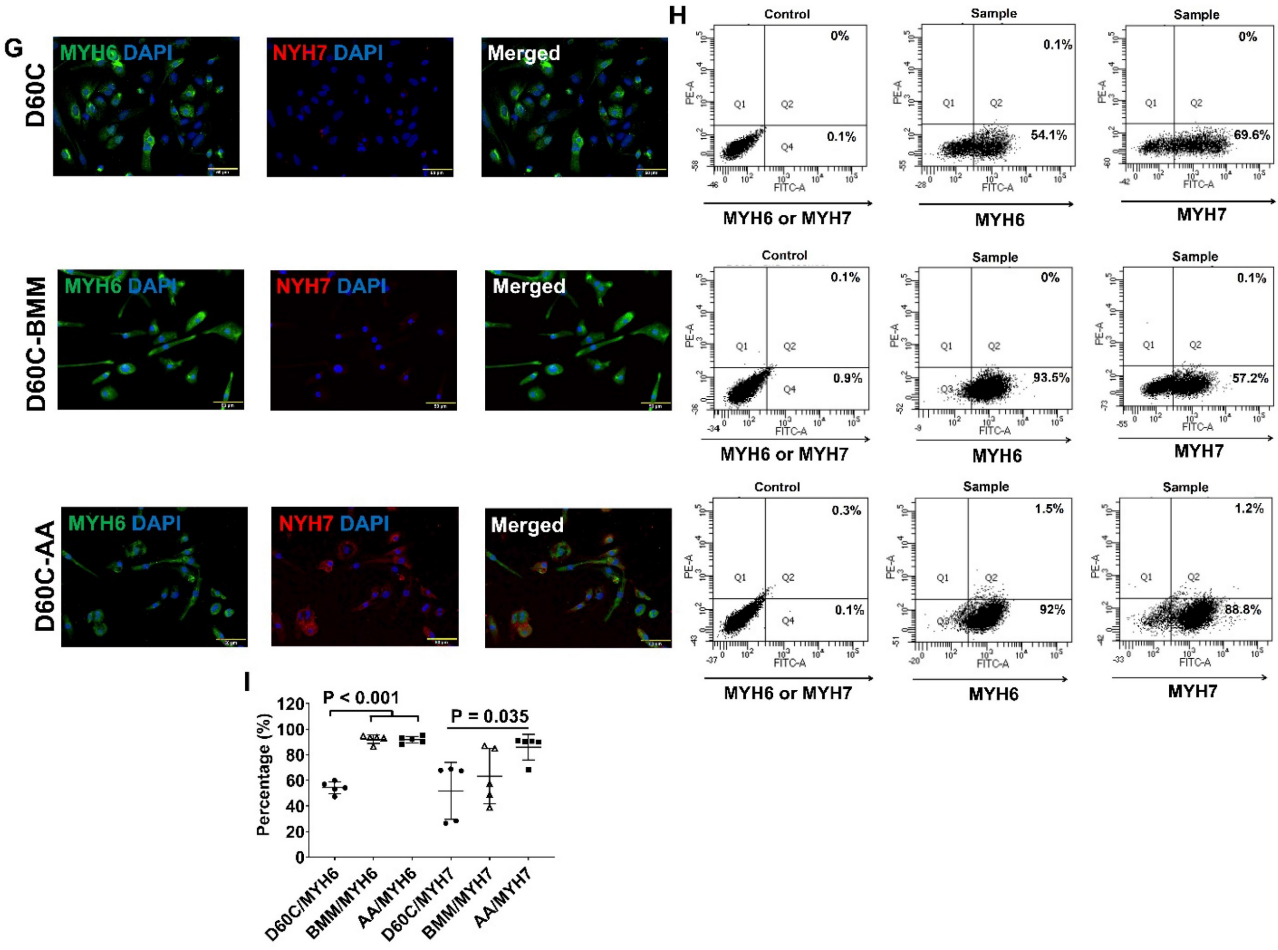

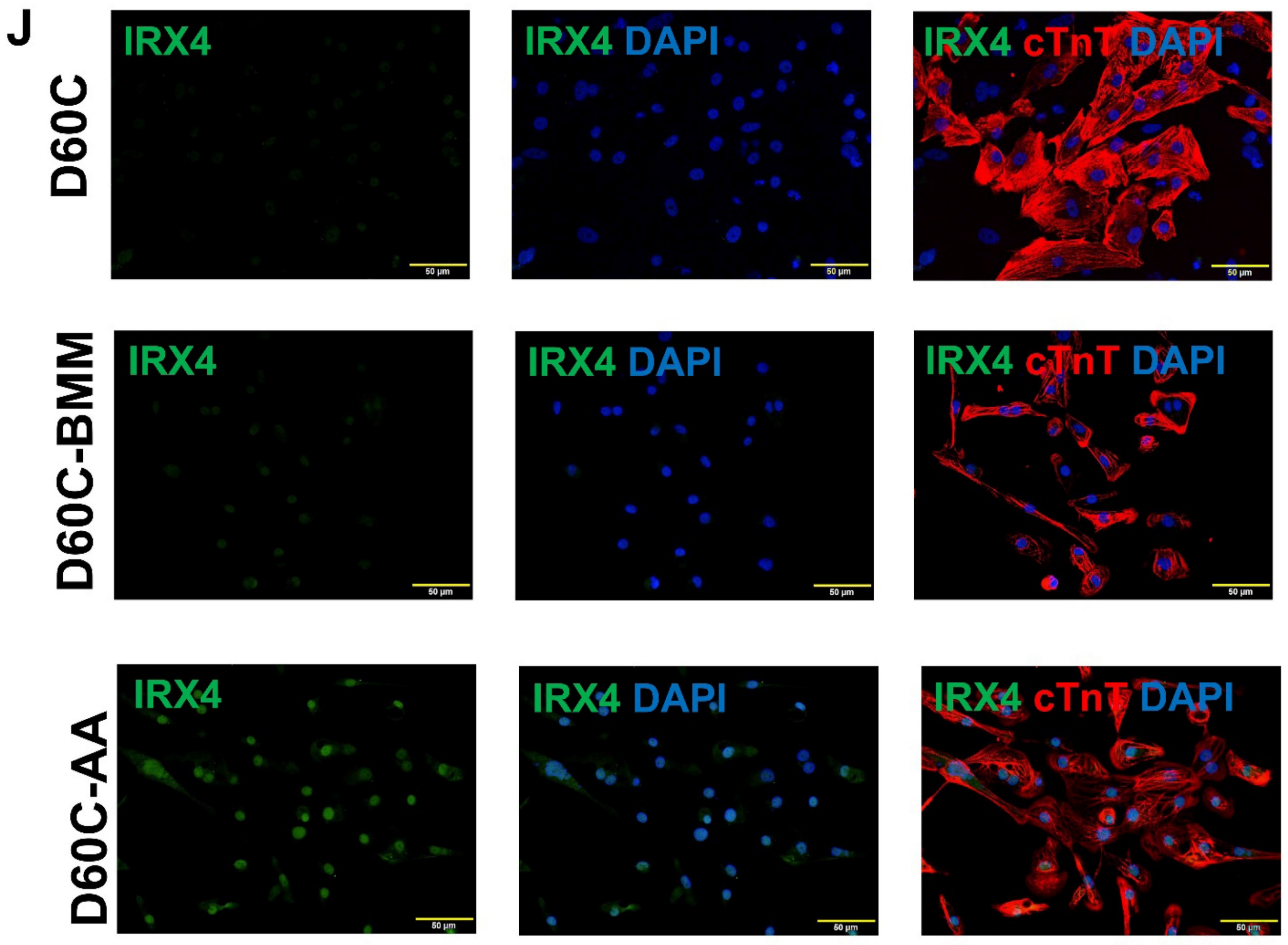
** Characterization of MLC2a, MLC2v, cTnT, TNNI3, MYH6, MYH7, and IRX4 protein expressions in D60C, BMM, or AA treated PCBC-CMs.** PCBC-CMs were maintained up to D60C, purified, and cultured in BMM (D60C-BMM) or BMM supplemented with AA (D60C-AA) for 14 days. Fluorescence immunostaining **(A)**, flow cytometry analysis** (B)**, and quantification** (C)** of D60C, BMM, or AA treated PCBC-CMs for MLC2a and MLC2v protein expressions. Fluorescence immunostaining **(D)**, flow cytometry analysis **(E)**, and quantification **(F)** of D60C, BMM, or AA treated PCBC-CMs for cTnT and TNNI3 protein expressions. Fluorescence immunostaining **(G)**, flow cytometry analysis **(H)**, and quantification** (I)** of D60C, BMM, or AA treated PCBC-CMs for MYH6 and MYH7 protein expressions.** (J)** Fluorescence immunostaining of D60C, BMM, or AA treated PCBC-CMs for IRX4 and cTnT protein expressions. (n = 5 each). Values are presented as the means ± SD. One-way ANOVA). (Bar = 50 μm).

Considering that D30C may not have subtype-specification completed, D60C PCBC-CMs were assessed. Both fluorescence immunostaining and FACS analysis showed that AA promoted abundant MLC2v protein expression in D60C-AA cells (Figure 7A-C). The cells expressing only MLC2a significantly reduced, while the cells expressing both MLC2a and MLC2V significantly increased and were higher than those of D60C and BMM treated cells (Figure 7C, p < 0.001 for all).

Furthermore, both fluorescence immunostaining and FACS analysis showed although AA promoted TNNI3 and MYH7 protein expressions, they were only significantly higher than that of D60C PCBC-CMs (Figure 7D-I). Fluorescence immunostaining showed that IRX4 protein expression was the strongest in AA treated PCBC-CMs (Figure 7J).

### AA promoted MLC2v, TNNI3, MYH7, and IRX4 protein expressions in RA induced atrial-like PCBC-CMs

**Figure 8:**
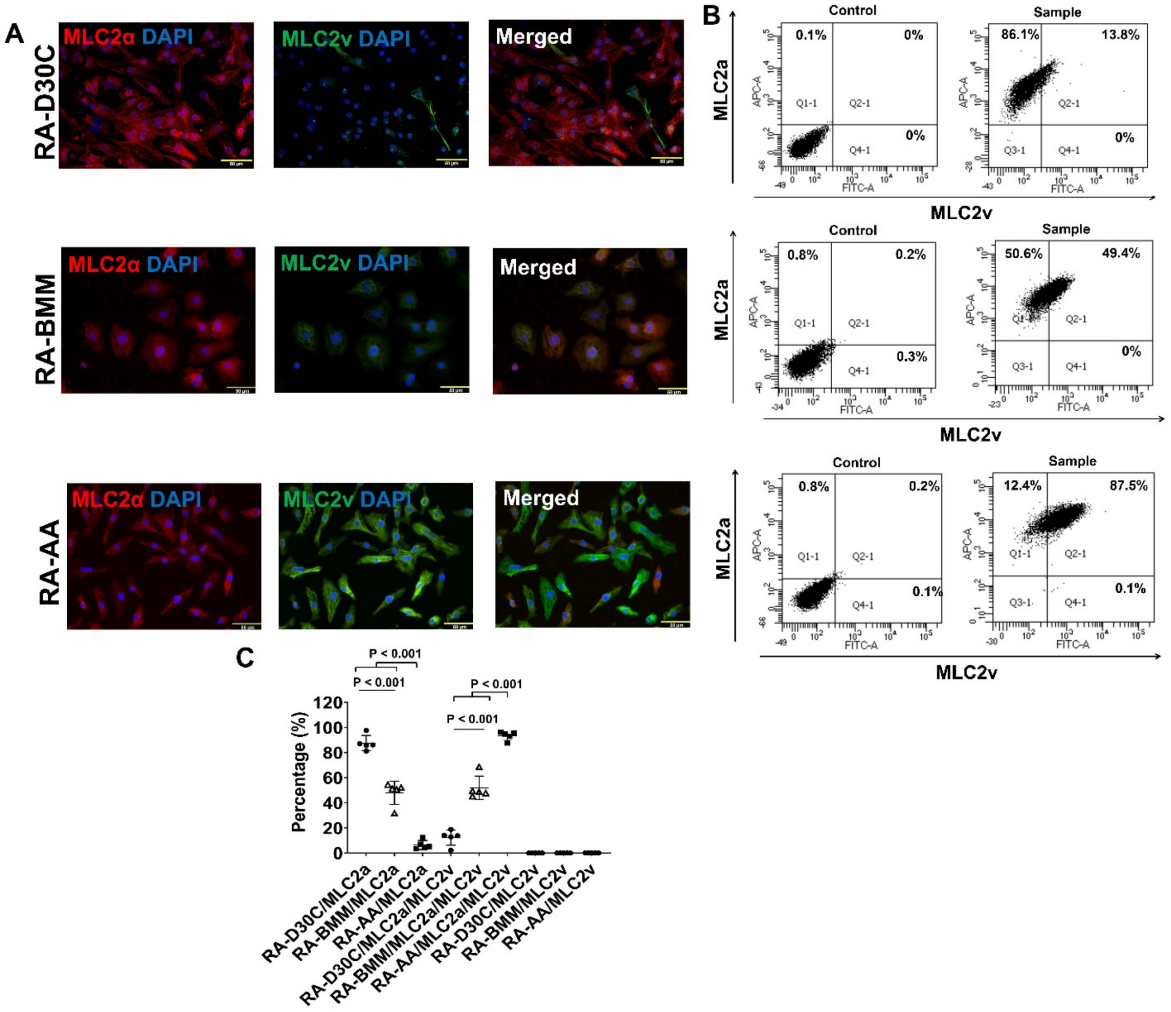

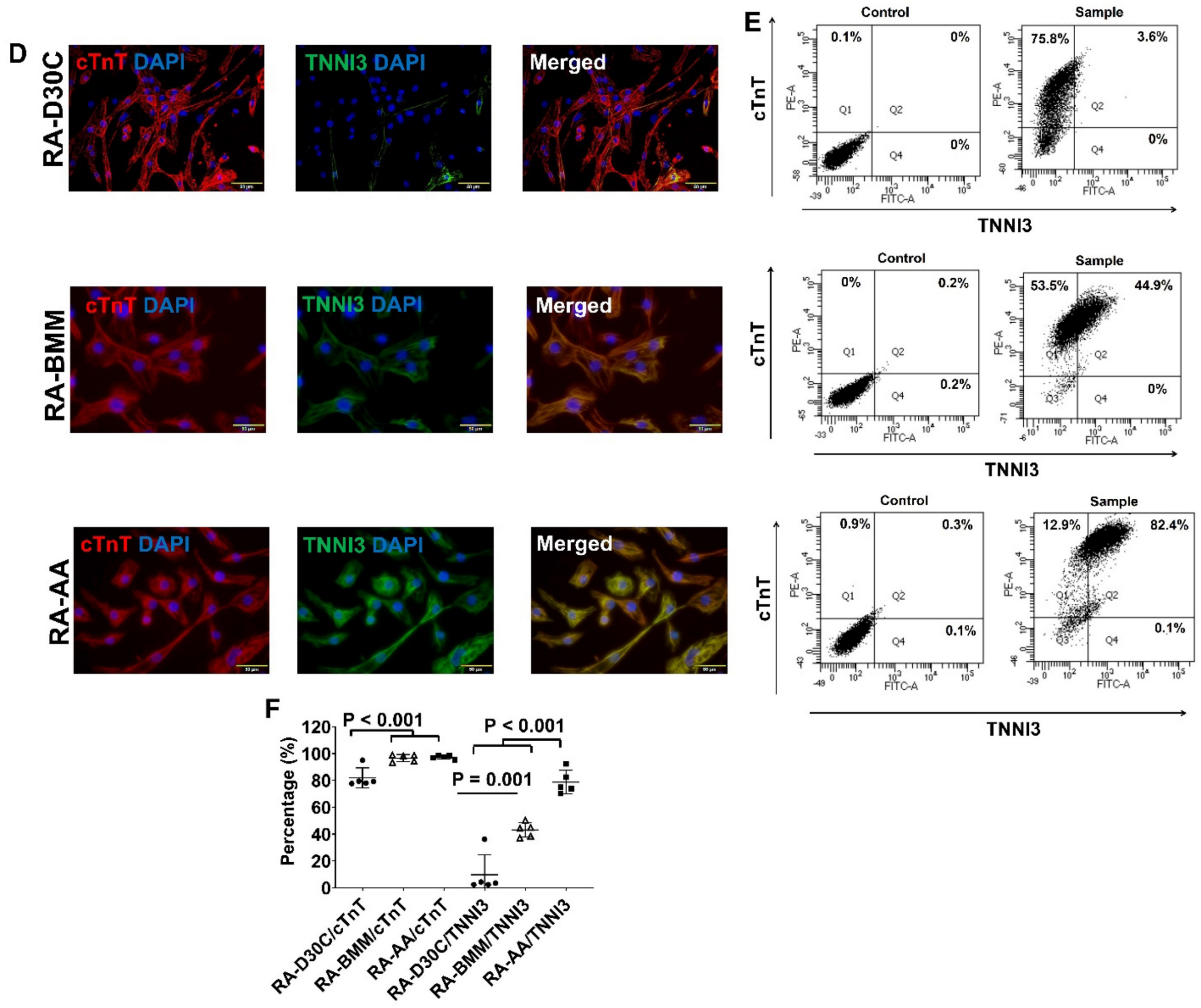

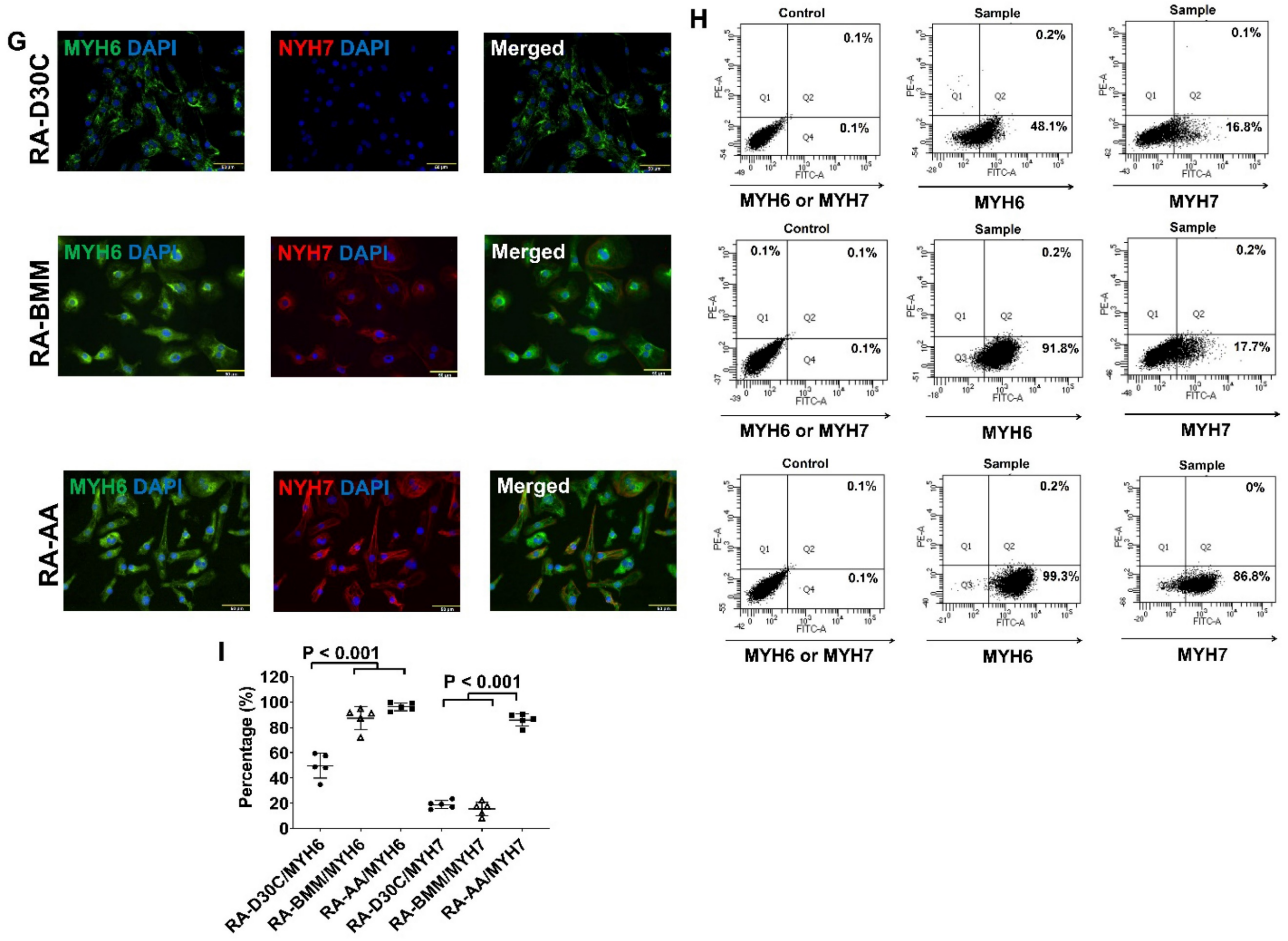

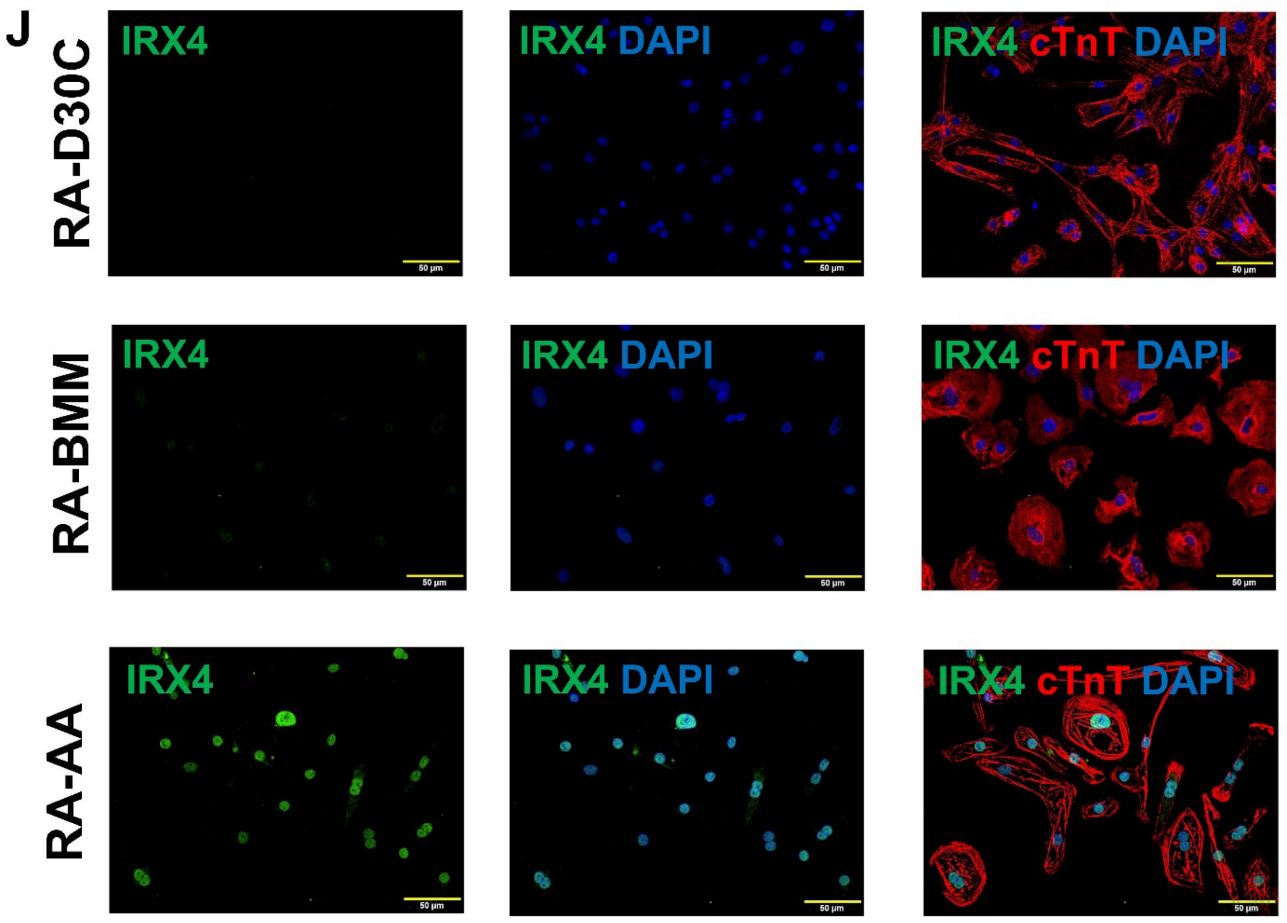
** Characterization of MLC2a, MLC2v, cTnT, TNNI3, MYH6, MYH7, and IRX4 protein expressions in retinoic acid induced atrial-like PCBC-CMs.** Retinoic acid (RA) was supplemented into hiPSC differentiation medium on days 3-7 to derive atrial-like PCBC-CMs. Cells were maintained up to D30C (RA-D30C), purified, and cultured in BMM (RA-BMM), or BMM supplemented with AA (RA-AA) for 14 days. Fluorescence immunostaining **(A)**, flow cytometry analysis **(B)**, and quantification **(C)** of D30C, BMM, or AA treated celsl for MLC2a and MLC2v protein expressions. Fluorescence immunostaining **(D)**, flow cytometry analysis** (E)**, and quantification **(F)** of D30C, BMM, or AA treated cells for cTnT and TNNI3 protein expressions. Fluorescence immunostaining **(G)**, flow cytometry analysis **(H)**, and quantification **(I)** of D30C, BMM, or AA treated cells for MYH6 and MYH7 protein expressions. **(J)** Fluorescence immunostaining of D30C, BMM, or AA treated cells for IRX4 and cTnT protein expressions. (n = 5 each). Values are presented as the means ± SD. One-way ANOVA). (Bar = 50 μm).

To determine the effect of AA on atrial-like hiPSC-CM, RA was supplemented in differentiation days 3-7 to induce atrial-like PCBC-CMs. Fluorescence immunostaining and FACS analysis showed that AA was able to promote MLC2v protein expression in RA induced atrial-like PCBC-CMs (Figure 8A-C). Similarly, the cells expressing only MLC2a significantly reduced, while the cells expressing both MLC2a and MLC2V significantly increased and were higher than those of RA-D30C or RA-BMM treated cells (Figure 8C, p < 0.001 for all).

Furthermore, both fluorescence immunostaining and FACS analysis showed that AA also promoted TNNI3 and MYH7 protein expressions which were significantly higher than those of RA-D30C and RA-BMM treated PCBC-CMs (Figure 8D-I). Fluorescence immunostaining showed that IRX4 protein expression was the strongest in AA treated RA-AA cells (Figure 8J).

### AA promoted MLC2v protein expressions in MLC2v-CMs

**Figure 9:**
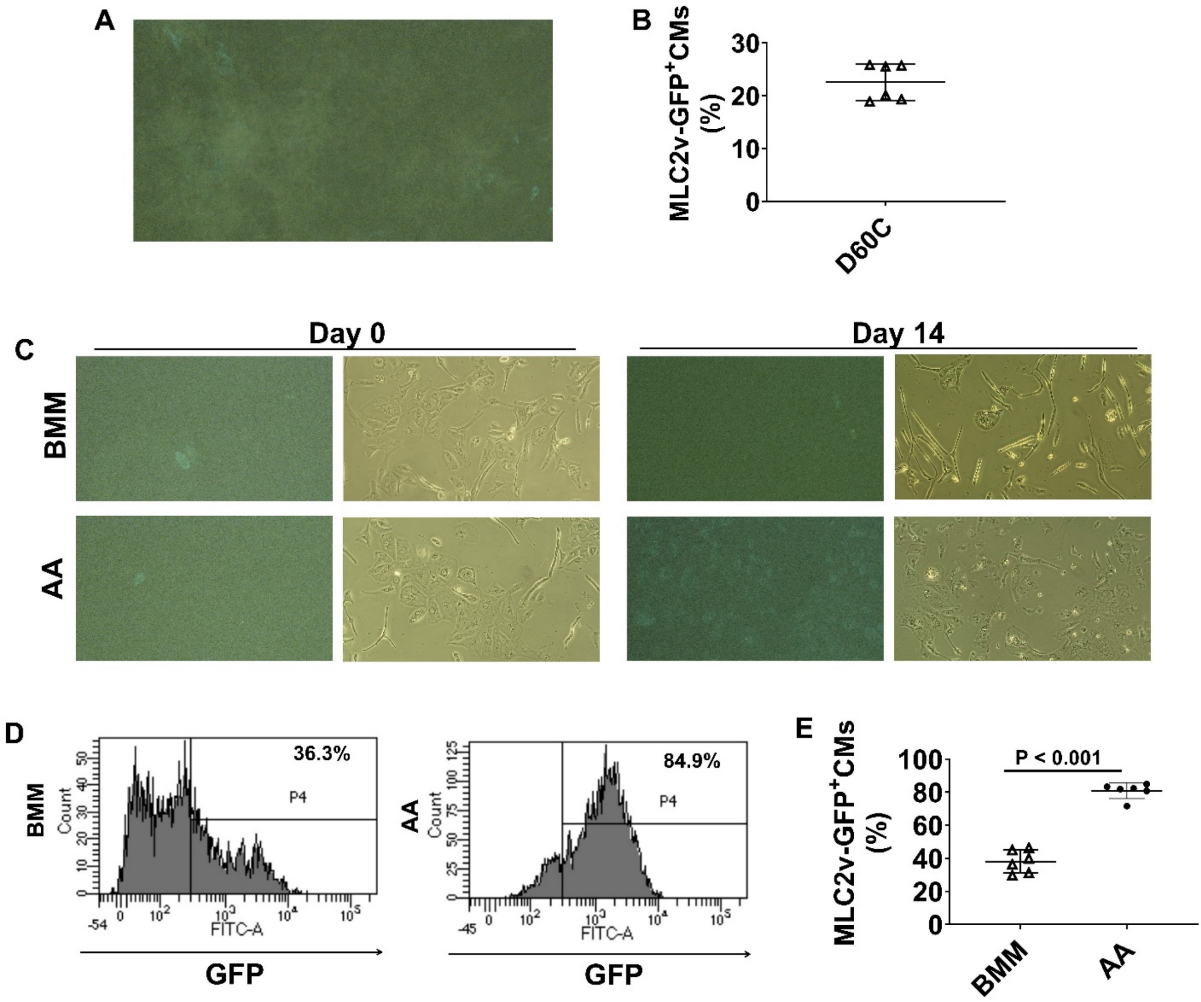
** Monitoring MLC2v protein expression using MLC2v-mEGFP hiPSC line**. **(A)** A representative image of GFP expressing MLC2v-hiPSC-CMs. **(B)** The mean percentage of MLC2v-CMs expressing GFP on D60C. **(C)** Representative fluorescence and corresponding phase contrast images of MLC2v-CMs on days 0 and 14 after BMM or AA treatment. **(D)** Representative flow cytometry images for determining GFP expressing in MLC2v-CMs. **(E)** The mean percentage of MLC2v-CMs expressing GFP. (n = 6 each). Values are presented as the means ± SD. Independent T-test).

To further confirm the findings, we employed a transgenic reporter hiPSC line, MLC2v-mEGFP hiPSC which has GFP-tagged MLC2v gene, to monitor GFP expression. MLC2v-CMs were cultured up to D60C (Figure 9A and videos 1 & 2) and FACS analysis showed that about 22.6% MLC2v-CMs expressed GFP (Figure 9B). When MLC2v-CMs were cultured in BMM only or BMM supplemented with AA, the GFP expressing MLC2v-CMs only slightly increased to 38% in BMM treated cells, while it significantly increased to 80.8% in AA treated cells (Figure 9C-E).

### Bobcat339 reduced MLC2v, TNNI3, MYH7, and IRX4 protein expressions in AA treated PCBC-CMs

**Figure 10:**
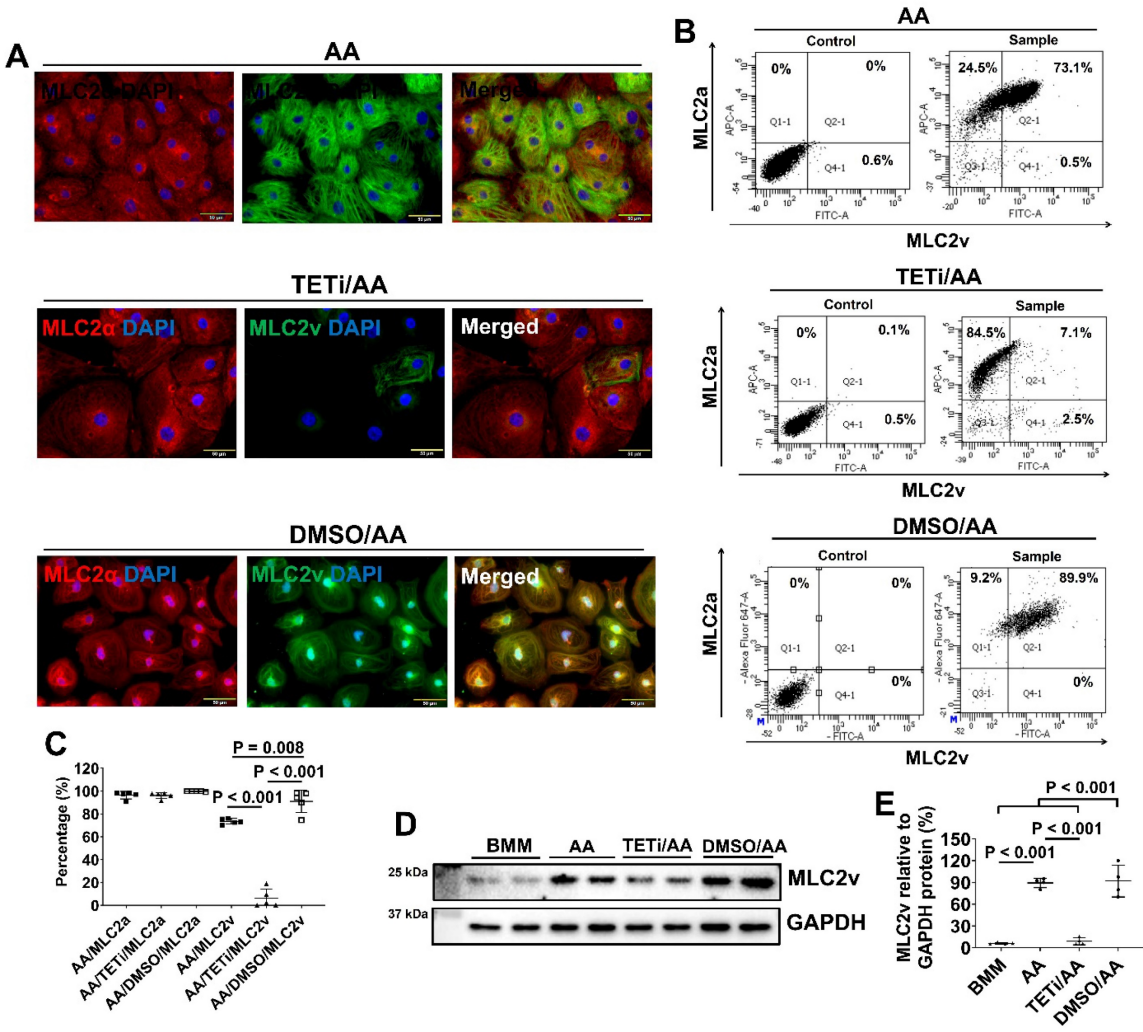
** The effect of Bobcat339 on the MLC2a and MLC2v protein expressions in PCBC-CMs. (A)** Fluorescence immunostaining of AA, TETi/AA, or DMSO/AA treated PCBC-CMs for MLC2a and MLC2v protein expressions. Flow cytometry analysis of AA, TETi/AA, or DMSO/AA treated PCBC-CMs for MLC2a and MLC2v protein expressions **(B)** and quantification **(C)**. Western Blot analysis of BMM, AA, TETi/AA, or DMSO/AA treated PCBC-CMs for MLC2v protein expression **(D)** and quantification **(E)**. (n = 5 each for panel C and n = 4 each for panel E. Values are presented as the means ± SD. One-way ANOVA). (Bar = 50 μm).

To investigate whether AA promoted MLC2v protein expression through DNA demethylation 40, PCBC-CMs were pretreated with Bobcat339, an inhibitor for TET enzyme in DNA demethylation, before AA treatment. Pretreatment with DMSO was used as a vehicle control. Fluorescence immunostaining showed that Bobcat339 substantially and significantly reduced MLC2v protein expression, while DMSO did not inhibit (Figure 10A). This was confirmed by FACS analysis showing that the MLC2v+ PCBC-CMs were reduced from 73.5 ± 2.5% (AA treatment) to 6.3 ± 8.1% (TETi/AA treatment), while it was 91.0 ± 9.8% after DMSO treatment (Figure 10B & C). Western Blot showed that Bobcat339 significantly reduced MLC2v protein expression after AA treatment, while DMSO had no inhibitory effect (Figure 10D & E).

**Figure 11:**
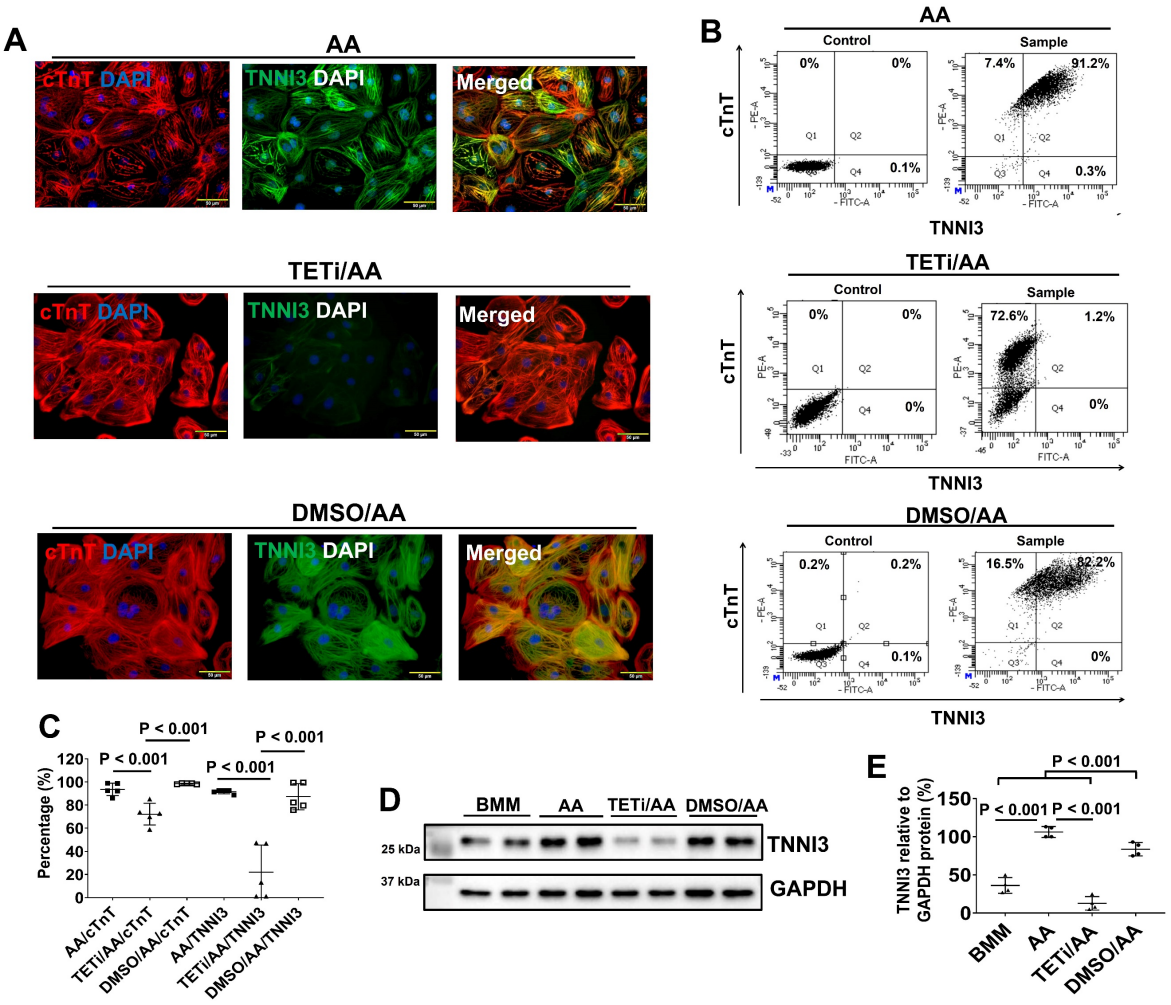
** The effect of Bobcat339 on the cTnT and TNNI3 protein expressions in PCBC-CMs. (A)** Fluorescence immunostaining of AA, TETi/AA, or DMSO/AA treated PCBC-CMs for cTnT and TNNI3 protein expressions. Flow cytometry analysis of AA, TETi/AA, or DMSO/AA treated PCBC-CMs for cTnT and TNNI3 protein expressions **(B)** and quantification **(C)**. Western Blot analysis of BMM, AA, TETi/AA, or DMSO/AA treated PCBC-CMs for TNNI3 protein expression **(D)** and quantification **(E)**. (n = 5 each for panel C and n = 4 each for panel E. Values are presented as the means ± SD. One-way ANOVA). (Bar = 50 μm).

Fluorescence immunostaining also showed that Bobcat339 significantly reduced TNNI3 protein expression in PCBC-CMs (Figure 11). FACS analysis confirmed that Bobcat339 significantly reduced both cTnT and TNNI3 protein expression in PCBC-CMs. More substantially, TNNI3+ hiPSC-CMs were reduced from 91.4 ± 1.7% (AA treatment) to 21.9 ± 23.4% (TETi/AA treatment), while it was 87.2 ± 11.3% after DMSO treatment (Figure 11B & C). Western Blot further confirmed that Bobcat339 significantly reduced TNNI3 protein expression (Figure 11D & E).

**Figure 12:**
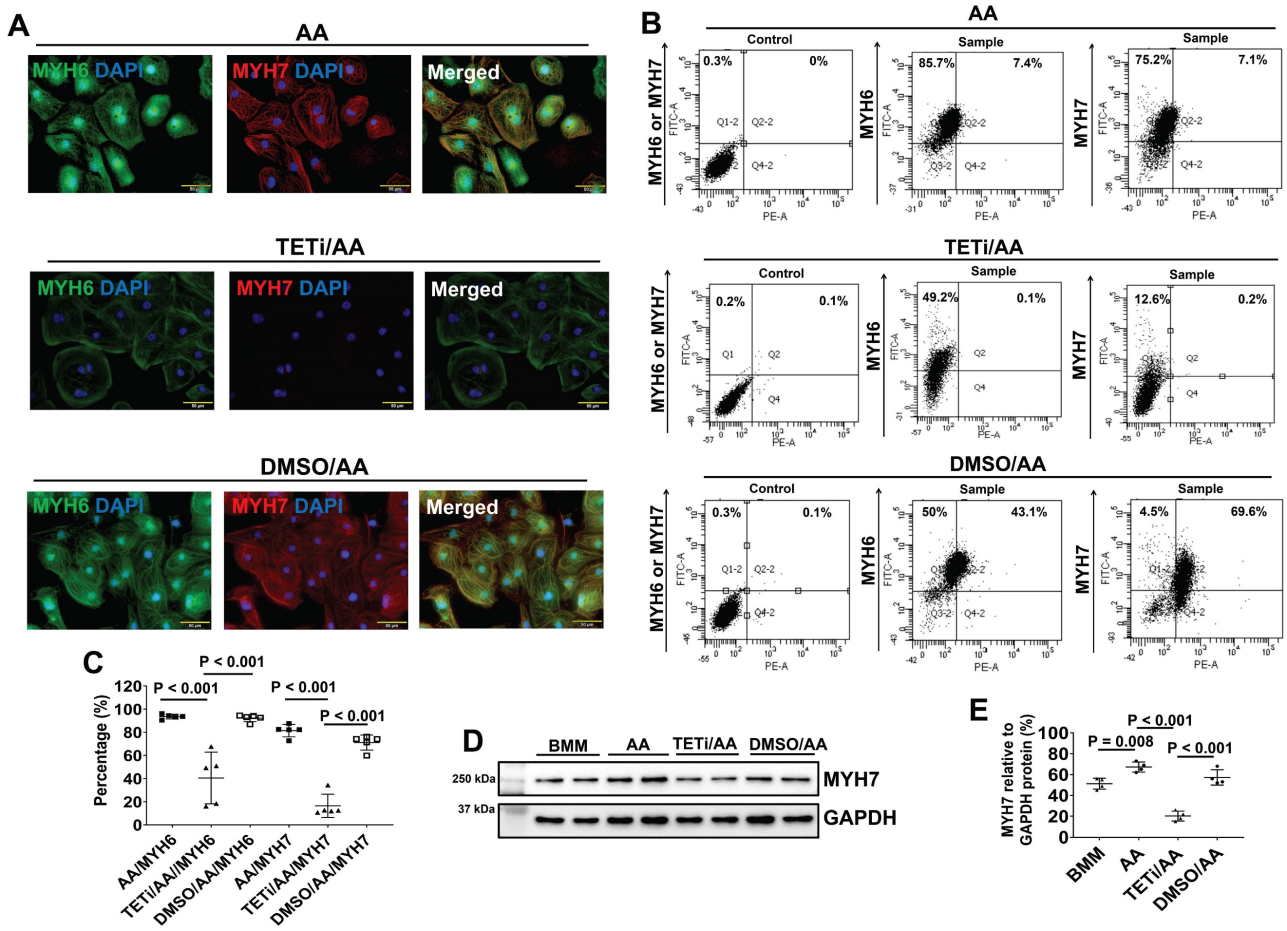
** The effect of Bobcat339 on the MYH6 and MYH7 protein expressions in PCBC-CMs. (A)** Fluorescence immunostaining of AA, TETi/AA, or DMSO/AA treated PCBC-CMs for MYH6 and MYH7 protein expressions. Flow cytometry analysis of AA, TETi/AA, or DMSO/AA treated PCBC-CMs for MYH6 and MYH7 protein expressions **(B)** and quantification **(C)**. Western Blot analysis of BMM, AA, TETi/AA, or DMSO/AA treated PCBC-CMs for MYH7 protein expression **(D)** and quantification **(E)**. (n = 5 each for panel C and n = 4 each for panel E. Values are presented as the means ± SD. One-way ANOVA). (Bar = 50 μm).

Furthermore, fluorescence immunostaining showed that Bobcat339 also significantly reduced MYH6 and MYH7 protein expression levels in PCBC-CMs (Figure 12A). FACS analysis also showed that Bobcat339 significantly reduced both MYH6 and MYH7 protein expressions in hiPSC-CMs. MYH7^+^PCBC-CMs reduced from 81.5 ± 5.4% (AA treatment) to 16.5 ± 10.1% (TETi/AA treatment), while it was 71.1 ± 6.5% after DMSO treatment (Figure 12B & C). Western Blot further confirmed that Bobcat339 significantly reduced MYH7 protein expression (Figure 12D & E).

**Figure 13:**
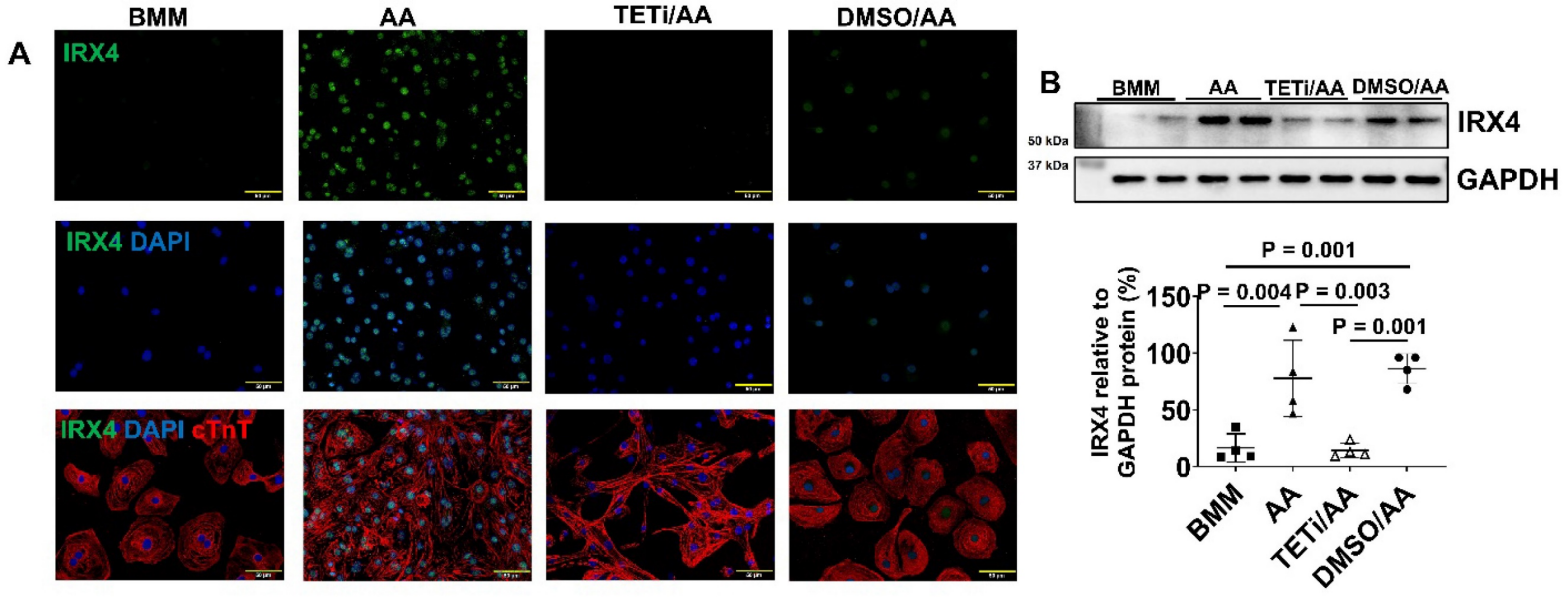
** The effect of Bobcat339 on IRX4 protein expression in PCBC-CMs**. Fluorescence immunostaining** (A)** and Western Blot analysis** (B)** of D30C, AA, TETi/AA, and DMSO/AA treated PCBC-CMs for IRX4 and cTnT protein expressions. (n = 4 each). Values are presented as the means ± SD. One-way ANOVA). (Bar = 50 μm).

Fluorescence immunostaining and Western Blot showed that Bobcat339 also substantially reduced IRX4 protein expression in AA treated PCBC-CMs (Figure 13A & B), suggesting that AA mediated MLC2v protein expression may also involve IRX4 induction.

### Bobcat339 inhibited SERCA2a protein expression in hiPSC-CMs after AA treatment

**Figure 14:**
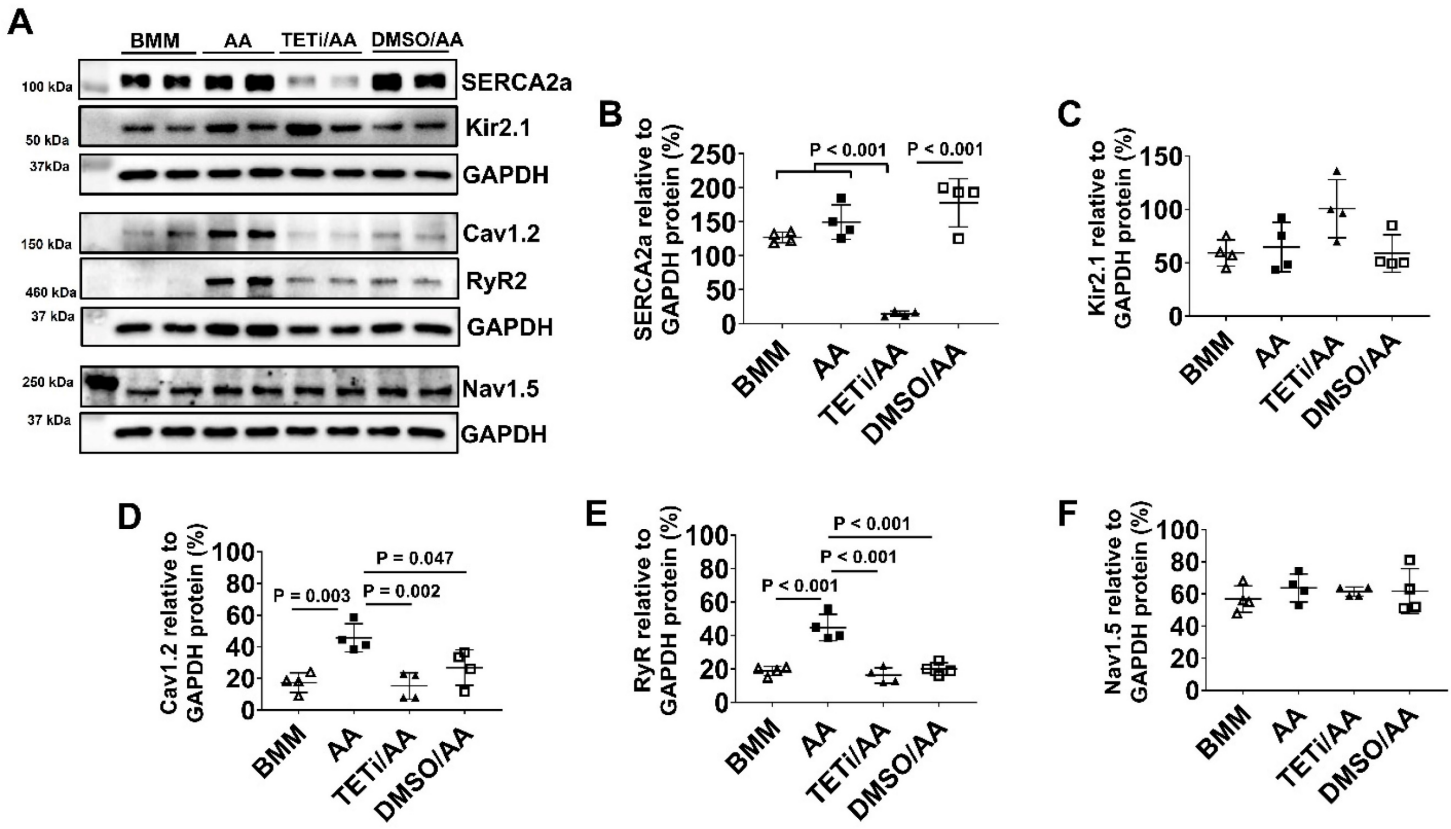
** The effect of Bobcat339 on the ion channel protein expressions in PCBC-CMs. (A)** Protein expression levels of SERCA2a, RyR2, Kir2.1, Nav1.5, and Cav1.2 in BMM, AA, TETi/AA, or DMSO/AA treated PCBC-CMs. Quantification of SERCA2a **(B)**, Kir2.1 **(C)**, Cav1.2 **(D)**, Ryr2 **(E)**, and Nav1.5 **(F)** protein expressions in BMM, AA, TETi/AA, or DMSO/AA treated PCBC-CMs. (n = 4 each). Values are presented as the means ± SD. One-way ANOVA). (Bar = 50 μm).

Western Blot showed that Bobcat339 significantly reduced SECA2a, Cav1.2, and RyR protein expression in PCBC-CMs, while it had no inhibitory effect on Kir2.1 and Nav1.5 protein expression (Figure 14). However, DMSO treatment also significantly reduced Cav1.2 and RyR protein expression, indicating that DMSO may have inhibitory effect on them which is independent of Bobcat339. Thus, the reduced Cav1.2 and RyR protein expressions after Bobcat339 treatment is at least partially due to DMSO.

### Bobcat339 decreased MFN-1 and increased FIS-1 protein expression in PCBC-CMs after AA treatment

**Figure 15:**
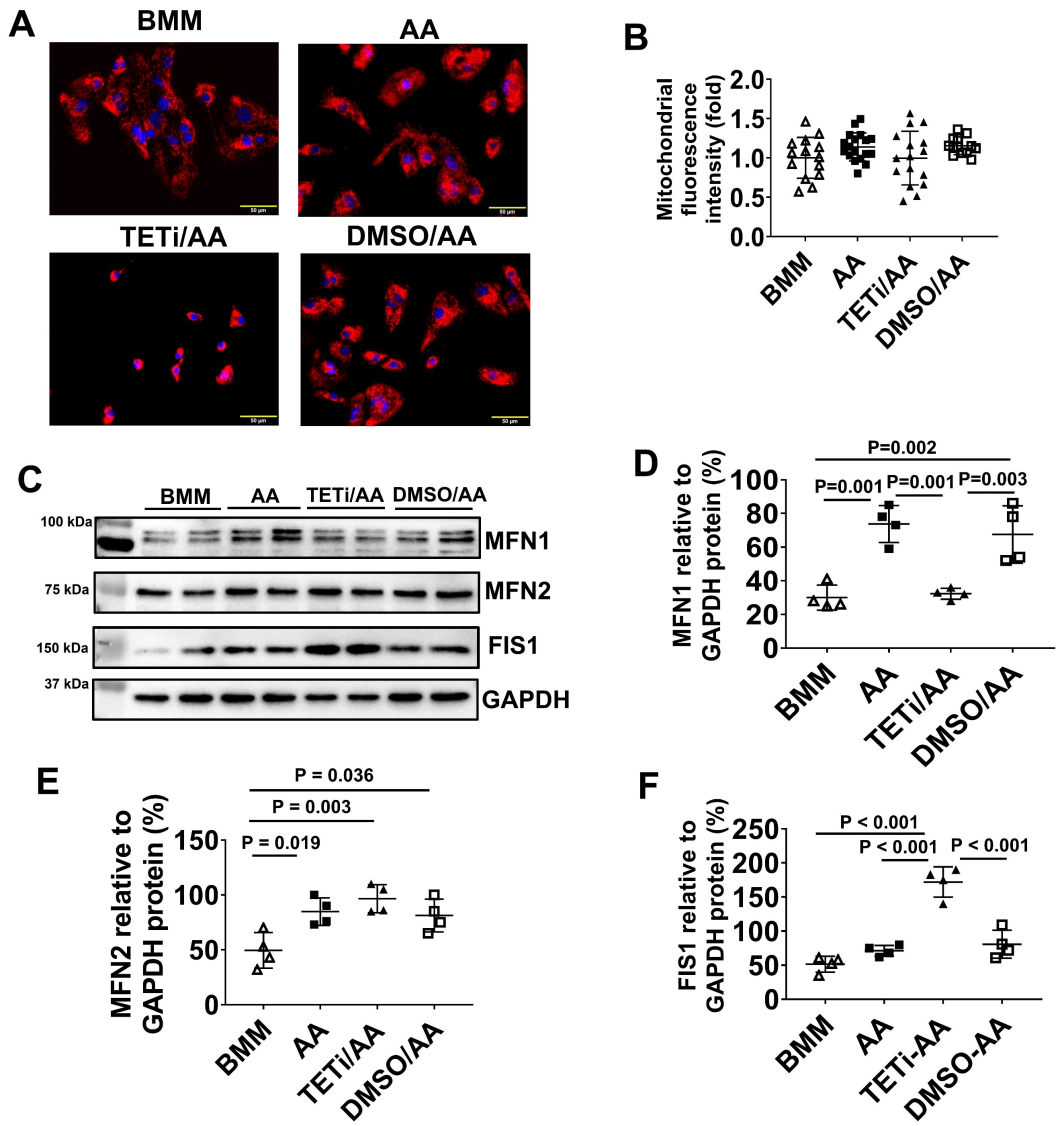
** The effect of Bobcat339 on the mitochondrial membrane potential in PCBC-CMs. (A)** JC-1 staining to show mitochondrial membrane potential.** (B)** Quantification of fluorescence intensity based on JC-1 staining. **(C)** Western Blot analysis for protein expressions of MFN1, MFN2, and FIS1. Quantification of MFN1 **(D)**, MFN2 **(E)**, and FIS1 **(F)** protein expressions in BMM, AA, TETi/AA, or DMSO/AA treated PCBC-CMs. (n = 14 - 18 for panel B and n = 4 each for panels D - F. Values are presented as the means ± SD. One-way ANOVA). (Bar = 50 μm).

JC-1 staining showed that although Bobcat339 did not significantly reduce mitochondrial mass and membrane potential based on fluorescence intensity, it caused mitochondria to cluster perinuclearly in PCBC-CMs (Figure 15A & B). Western Blot showed that Bobcat339 significantly reduced MFN1 and increased FIS1 protein expression in PCBC-CM, while it had no effect on MFN2 protein expression (Figure 15C - F). These results indicate that mitochondrial fission is enhanced after DNA demethylation is inhibited.

### Bobcat339 inhibited DNA demethylation induced by AA through inhibiting TET1 and TET2 protein expression

**Figure 16:**
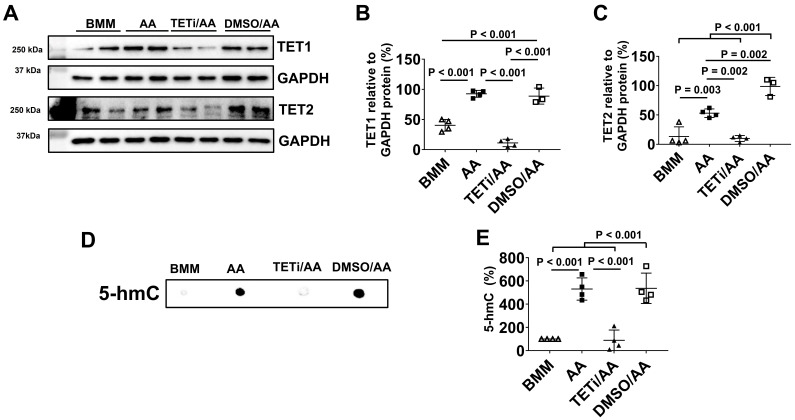
** The effect of Bobcat339 on TET1 and TET2 protein expression in PCBC-CMs. (A)** Protein expressions of TET1 and TET2 in BMM, AA, TETi/AA, or DMSO/AA treated PCBC-CMs. Quantification of TET1 **(B)** and TET2** (C)** protein expressions. DNA dot plot analysis **(D)** and quantification **(E)** for 5-hmC expression in BMM, AA, TETi/AA, or DMSO/AA treated PCBC-CMs. (n = 3 - 4 for panels B & C and n = 4 each for panel E). Values are presented as the means ± SD. One-way ANOVA). (Bar = 50 μm).

As Bobcat339 is an inhibitor of TET1 and TET2, Western Blot was performed to assess whether Bobcat339 could inhibit TET1 and TET2 protein expressions. Pretreatment with Bobcat339 inhibited TET1 and TET2 protein expressions induced by AA (Figure 16A-C). DMSO did not inhibit TET1 and TET2 protein expression. In fact, DMSO significantly increased TET2 protein expression as compared with AA treatment.

As 5-hmC is the first oxidative product of the 5-methylcytosine (5-mC) demethylation by TET oxidization, DNA dot plot was employed to determine 5-hmC content in DNA of PCBC-CMs (Figure 16D). DNA dot plot showed that AA significantly up-regulated 5-hmC product which was inhibited by Bobcat339 and DMSO had no inhibitory effect on 5-hmC product (Figure 16D & E). These results suggest that AA may act through TET1/TET2 to promote DNA demethylation of PCBC-CMs which then helps convert atrial-like hiPSC-CMs to ventricular-like hiPSC-CMs *in vitro*.

### AA increased MLC2v protein expression, mitochondrial membrane potential and mass, and promoted ventricular-like CMs in DP3-CMs

To determine whether the effect of AA on inducing MLC2v expression and promoting ventricular-like hiPSC-CMs is limited on PCBC-CMs, AA was applied to a disease hiPSC line. DP3-hiPSC is a disease hiPSC line which was reprogrammed from dermal fibroblasts of patients with type 2 diabetes 28, 29. Similar to the results with PCBC-CM, AA significantly increased MLC2v protein expression in DP3-CMs Figure S5. Both fluorescence immunostaining and FACS analysis showed that AA promoted abundant MLC2v protein expression in DP3-CMs. About 86.7 ± 4.3% of DP3-CMs expressed MLC2v after AA treatment, which was significantly higher than those in D30C or BMM treated cells (p < 0.001 for both).

Both fluorescence immunostaining and FACS analysis showed that BMM or AA treatment significantly increased TNNI3 protein expression as compared with D30C DP3-CMs Figure S6. Although there is a trend of increased TNNI3 protein expression, AA did not further significantly increase TNNI3 protein expression as compared with BMM treatment.

Both BMM and AA treatment significantly increased MYH7 protein expression as compared with D30C DP3-CMs as shown by fluorescence immunostaining and FACS analysis Figure S7. AA further significantly increase MYH7 protein expression as compared with BMM treated cells as determined by FACS analysis.

JC-1 staining showed that although both BMM and AA treated DP3-CMs had significantly increased fluorescence intensity as compared with D30C cells, AA treatment resulted in the highest fluorescence intensity which was significantly higher than BMM treated DP3-CMs. Mitochondria were evenly distributed in the cytoplasm of BMM or AA treated DP3-CMs Figure S8.

Metabolomic profiling showed that BMM treatment increased C16 and C18:1 acylcarnitine content in DP3-CMs. However, AA significantly increased both C3 and C5 as well as C16 and C18:1 acylcarnitine content in DP3-CMs as compared with D30C cells Figure S8E & F).

Patch clamp studies showed that 72.4% of D30C DP3-CMs had atrial-like action potential and only 27.6% of them showed ventricular-like action potential. AA treatment significantly increased ventricular-like DP3-CMs to 87.5% (p < 0.001, Figure S9A & B). The longest ADP80 was found in AA treated ventricular-like DP3-CMs which was significantly longer than D30C atrial-like cells (p = 0.007) or AA treated atrial-like cells (p = 0.001) (Figure S9C).

When Bobcat339 was applied to DP3-CMs before AA treatment, both fluorescence immunostaining and FACS analysis showed that Bobcat339 only significantly reduced MLC2v protein expression in DP3-CMs treated with AA (Figure S10A, B, and M). The MLC2v^+^ DP3-CMs significantly reduced from 86.7 ± 4.3% (AA treatment) to 14.1 ± 18% (TETi/AA treatment). Although, there are trends of reduced TNNI3 and MYH7 protein expression in DP3-CMs after Bobcat339 treatment, no significant difference was found between AA and TETi/AA treatments (Figure S10B-F, N, and O). DMSO had no inhibitory effect on MLC2v, TNNI3, and MYH7 protein expression in DP3-CMs (Figure S10G-L, N, and O). In fact, it enhanced TNNI3 protein expression in DP3-CMs.

DNA dot plot of DP3-CMs showed that AA significantly up-regulated 5-hmC product which was inhibited by Bobcat339, while DMSO did not inhibit 5-hmC product (Figure S11. This confirms the same finding for PCBC-CMs.

## Discussion

In the present study, we showed that AA induced MLC2v protein expression promoted ventricular-like CMs in hiPSC-CMs through DNA demethylation. When DNA demethylation was inhibited by Bobcat339, MLC2v protein expression was dramatically reduced, indicating that AA induced DNA demethylation is at least partially involved in promoting MLC2v protein expression.

hiPSCs, like embryonic stem cells (ESCs), have the ability to proliferate indefinitely yet retain the ability to differentiate into any cell of the body including CMs. Validated protocols for the reliable and high efficiency differentiation of hiPSCs and hESCs into CMs has not only opened the doors for hiPSC-CMs transfer therapy 7, 8, but also can serve as disease models for novel drug development.

The newly differentiated hiPSC-CMs are dominantly expressing MLC2a and immature. Although both the matrix sandwich protocol 2 and GiWi small molecule protocol 30 can efficiently differentiate hiPSCs into CMs, the derived hiPSC-CMs had only about 20% on day-30 or < 30% on day-40 expressed MLC2v, respectively. We and others have documented similar findings and the percentage of hPSC-CM expressing MLC2v protein was between 13-36% 7, 8, 41. Immature hPSC-CMs seem to be more proarrhythmic than mature cells 42. Thus, transfer of immature hPSC-CMs for myocardial repair may result in mismatched electrophysiology with the adult host's cardiac system, resulting in arrhythmias 43. The maturity of CMs is critical during drug development. Genotypically and phenotypically matured hPSC-CMs are needed for cost-effective screening and discovering drugs 44, 45.

Long-term culture 12, 13, drug selection, metabolism 16, 17, mechanical stimulus 46, 47, tissue engineering 14, 15, electrical stimulus 48, microRNA 49, cell-cell interaction 15, growth hormones 50, 51, and modulation of signaling pathways, such as mTOR 52 and WNT 53, have been shown to more or less efficiently promote maturation of hPSC-CMs. In the current study, we showed that AA, which is also called vitamin C and is an essential nutrient, promoted MLC2v protein expression in hiPSC-CMs. We systematically examined cell phenotype, including contractile protein expression, electrophysiology, and metabolism, between AA treated and non-treated hiPSC-CMs.

Firstly, we examined protein expression levels of MLC2v, TNNI3, and MYH7, which are predominantly expressed in mature or adult ventricular CMs 10, 54. AA promoted MLC2v protein expression in PCBC-CMs, DP3-CMs, MLC2v-CMs, and even in RA induced atrial PCBC-CMs. The higher abundance of sarcomeric protein expressions are expected to enhance contractility in AA treated hiPSC-CMs. However, we did not measure the single hiPSC-CM or engineered cardiac tissues, which is a limitation of this study.

Secondly, we determined ion channel protein expression levels. AA promoted RyR and Cav1.2 ion channel protein expression, but did not change the protein expression levels of SERCA2a (mediates Ca^2+^ reuptake into the sarcoplasmic reticulum), Kir2.1 (potassium channel), Nav1.5 (a sodium channel subunit). Ryanodine receptors (RyRs) are intracellular calcium channels that are responsible for the release of Ca^2+^ from the sarco/endoplasmic reticulum while Ca_v_1.2 is a subunit of L-type voltage-dependent calcium channel that mediates the influx of calcium ions into the CMs. Both proteins are involved in excitation-contraction of CMs.

Thirdly, we looked at mitochondrial membrane potential and mass. JC-1 staining showed that AA increased mitochondrial membrane potential and mass as indicated by higher fluorescence intensity and Western Blot analysis showed increased MFN2 protein expression, suggesting increased biogenesis. Then, we examined acylcarnitine profile and found that both BMM and AA significantly increase branched-chain amino acid (C3+C5) and fatty acid (C16+C18:1) metabolism. However, no difference was found between BMM and AA treated CMs. The significantly increased catabolism of branched-chain amino acids and long-chain fatty acids could be due to the lack of glucose in BMM, which forced hiPSC-CMs to use amino acids and fatty acids as fuels for metabolism. These data suggest that although AA promoted mitochondrial activity and biogenesis, it probably has no effect on branched-chain amino acid and fatty acid metabolism.

Next, we examined the electrophysiological characteristics of AA treated and non-treated PCBC-CMs and DP3-CMs. Initially only 20% and 27.6% of the D30C PCBC-CMs and DP3-CM, showed ventricular-like action potential. A 2-week AA treatment significantly increased the frequency of cells displaying ventricular-like action potential to 78.8% and 86.4%, respectively. Although the ventricular-like hiPSC-CMs in both cell lines appeared to have longer ADP80s, they were only longer than that of atrial-like hiPSC-CMs and had no difference compared with D30C ventricular-like hiPSC-CMs. These data suggest that although AA promoted the conversion of atrial-like hiPSC-CMs to ventricular-like hiPSC-CM, it does not improve electrophysiological maturation after 2-week's AA treatment.

Lastly, we explored the underlying mechanism through which AA promoted MLC2v protein expression. We found that AA promoted TET1 and TET2 protein expression, while Bobcat339, a potent and selective cytosine-based inhibitor of TET1 and TET2 enzymes, inhibited MLC2v protein expression in both PCBC-CMs and DP3-CMs after AA treatment. Bobcat339 inhibited not only 5-hmC, an oxidized product of TET1/TET2 activity, but also inhibited TNNI3, MYH7, SERCA2a, Cav1.2, RyR2, and MFN1, and increased FIS1 protein expressions in PCBC-CMs. These data suggest that AA promoted MLC2v protein expression at least partially through TET1/TET2 mediated DNA demethylation. Future studies are needed to explore how AA induces TET expression and the DNA methylation status upon AA or Bobcat339/AA treatment.

Interestingly, although DMSO, a solvent of Bobcat339, has no inhibitory effect on contractile protein expression, it can inhibit Cav1.2 and RyR protein expression. Thus, the reduced Cav1.2 and RyR protein expression in hiPSC-CMs could be due to either TETi, or DMSO, or both after treated with TETi and AA.

In the current study, 3 hiPSC lines were used. All cell line derived CMs had increased MLC2v protein expression in hiPSC-CMs. However, the disease cell line, DP3-CMs had different phenotypes as compared with PCBC-CMs in response to AA treatment. AA increased TNNI3 and MYH7 protein expression in PCBC-CMs, but it did not increase them in DP3-CMs. AA further increased mitochondrial membrane potential and mass in DP3-CM, but it did not increase them in PCBC-CMs. This may be due to the disease associated with DP3-CMs. Type 2 diabetes has dysregulated metabolism and altered mitochondrial function. AA treatment of DP3-CMs may help overcome diabetes-related defects in mitochondrial function and promote mitochondrial biogenesis.

In conclusion, we found that AA induced MLC2v protein expression and promoted ventricular-like CM subtype in hiPSC-CMs. AA promoted MLC2v protein expression is at least partially through DNA demethylation mediated by TET1/TET2. Using AA may be a cost-effective method to promote ventricular-like hiPSC-CMs.

## Supplementary Material

Supplementary figures.

Supplementary video 1: contracting MLC2v-CMs on D60C.

Supplementary video 2: the same contracting MLC2v-CM area as Video-1 visualized under bright field.

## Figures and Tables

**Table 1:** Purification medium

Component	Dilution Factor	E.g. 500mL
RPMI no glucose	-	482.5mL
β-mercap (Cell culture grade)	1000x	500 μL
NEAA	100x	5 mL
GlutaMAX	100x	5 mL
Anti-Anti	100x	5 mL
1M Lactic Acid	250 x (4mM)	2 mL

**Table 2:** Basal maturation medium

Component	Dilution Factor	E.g. 500mL
RPMI no glucose	-	474.5 mL
β-mercap (Cell culture grade)	1000 x	500 μL
NEAA	100 x	5 mL
GlutaMAX	100 x	5 mL
Anti-Anti	100 x	5 mL
B27	50 x	10 mL

**Table 3:** Antibodies used for immunostaining

Primary antibody	Dilution	Category NO.	Company	2nd antibody	Dilution	Category NO.	Company
Mouse anti-myosin light chain atrial isoform	1:200	311011AT1	Synaptic systems	Goat anti-mouse IgG-PE	1:400	A31570	Thermo Fisher
Rabbit anti-myosin light chain ventricular isoform	1:200	10906-1-AP	Proteintech	Goat anti-rabbit IgG-FITC	1:400	A32790	Thermo Fisher
Mouse anti-cardiac troponin T-PE	1:20	564767	BD Biosciences	Goat anti-mouse IgG-PE	1:400	A31570	Thermo Fisher
Rabbit anti-troponin I3 (cardiac type)	1:200	ab-47003	Abcam	Goat anti-rabbit IgG-FITC	1:400	A32790	Thermo Fisher
Rabbit anti-myosin Heavy Chain 6	1:200	22281-1-AP	Proteintech	Goat anti-rabbit IgG-FITC	1:400	A32790	Thermo Fisher
Mouse anti-myosin Heavy Chain 7	1:10	MAB1548	Millipore	Goat anti-mouse IgG-PE	1:400	A31570	Thermo Fisher
Rabbit anti-IRX4	1:200	104135-T34	Sino Biological	Donkey anti-rabbit IgG-FITC	1:400	711-095-152	Jackson ImmunoResearch

**Table 4:** Antibodies used for fluorescence activated cell sorting

Primary antibody	Dilution	Category NO.	Company	2nd antibody	Dilution	Category NO.	Company
Mouse anti-myosin light chain atrial isoform-APC	1:600	311011AT1	Synaptic systems				
Rabbit anti-myosin light chain ventricular isoform	1:600	10906-1-AP	Proteintech	Goat anti-rabbit IgG-FITC	1:800	A32790	Thermo Fisher
Mouse anti-cardiac troponin T-PE	1:20	564767	BD Biosciences				
Rabbit anti-troponin I3 (cardiac type)	1:600	ab-47003	Abcam	Goat anti-rabbit IgG-FITC	1:1000	A32790	Thermo Fisher
Rabbit anti-myosin Heavy Chain 6	1:600	22281-1-AP	Proteintech	Goat anti-rabbit IgG-FITC	1:1000	A32790	Thermo Fisher
Rabbit anti-myosin Heavy Chain 7	1:600	MAB90961	Millipore	Goat anti-mouse IgG-FITC	1:1000	A32790	Thermo Fisher

**Table 5:** qRT-PCR primers

Gene Name	Forward	Reverse	Amplication size (bp)
GAPDH	TCGACAGTCAGCCGCATCTTCTTT	ACCAAATCCGTTGACTCCGACCTT	94
cTnT	GGAGAGAGAGTGGACTTTGATG	CCTCCTCTTTCTTCCTGTTCTC	109
TNNI3	GACAAGGTGGATGAAGAGAGATAC	CTTGCCTCGAAGGTCAAAGA	102
TNNI1	CTTTAGGGCGTGGGTCTTATC	TCTGTTCCCATTCATGTGTCTC	101
MYH6	CACCAACAATCCCTACGACTAC	AGCACGTCAAAGGCACTATC	108
MYH7	CTCGCCAGAATGGAGTACAAA	CTTCATCCAGGGCCAATTCT	108
MLC2a	CGTGGTTCTTCCAACGTCTT	CCATCACGATTCTGGTCGATAC	92
MYL2v	CCCATTTATCCACCTCCATCTT	TACACGACCTCCTGTTTATTGG	118
SLN	CTTGGTGTGCCCTCAGAAAT	CCTCACAAGGAGCCACATAAG	133

**Table 6:** Antibodies used in Western Blot analysis

Primary antibody	Dilution	Category NO.	Company	2nd antibody	Dilution	Company
Rabbit anti-myosin light chain ventricular isoform	1:500	10906-1-AP	Proteintech	Goat anti-rabbit-IgG-HRP	1:4000	Cell signaling
Rabbit anti-troponin I3 (cardiac type)	1:1000	ab-47003	Abcam	Goat anti-rabbit-IgG-HRP	1:4000	Cell signaling
Mouse-anti-myosin Heavy Chain 7	1:200	MAB1548	Millipore	Goat anti-mouse-IgG-HRP	1:4000	Cell signaling
Mouse anti-SERCA2a	1:1000	SC-376235	Santa Cruz	Goat anti-mouse-IgG-HRP	1:4000	Cell signaling
Mouse anti-RyR	1:200	SC-376507	Santa Cruz	Goat anti-mouse-IgG-HRP	1:1000	Cell signaling
Rabbit anti-Kir2.1	1:500	AB5374	Millipore	Goat anti-rabbit-IgG-HRP	1:2000	Cell signaling
Mouse Nav1.5	1:100	sc-81631	Santa Cruz	Goat anti-mouse-IgG-HRP	1:1000	Cell signaling
Mouse-Cav1.2	1:400	sc-398433	Santa Cruz	Goat anti-mouse-IgG-HRP	1:2000	Cell signaling
Mouse anti-MFN1	1:500	sc-166644	Santa Cruz	Goat anti-mouse-IgG-HRP	1:4000	Cell signaling
Mouse anti-MFN2	1:500	sc-100560	Santa Cruz	Goat anti-mouse-IgG-HRP	1:4000	Cell signaling
Mouse anti-Fis1	1:500	sc-376469	Santa Cruz	Goat anti-mouse-IgG-HRP	1:4000	Cell signaling
Rabbit anti-GAPDH	1:5000	2118S	Cell signaling	Goat anti-rabbit-IgG-HRP	1:5000	Cell signaling
Mouse anti-TET1	1:200	sc-293186	Santa Cruz	Goat anti-mouse-IgG-HRP	1:1000	Cell signaling
Rabbit anti-TET2	1:200	GTX124205	Gene Tex	Goat anti-rabbit-IgG-HRP	1:2000	Cell signaling
Rabbit anti-IRX4	1:500	104135-T34	Sino Biological	Goat anti-rabbit-IgG-HRP	1:4000	Cell signaling
