# Supplementary material for: Ascorbic acid induces MLC2v protein expression and promotes ventricular-like cardiomyocyte subtype in human induced pluripotent stem cells derived cardiomyocytes
Source: Theranostics. 2023 Jul 3;13(11):3872–96. doi: 10.7150/thno.80801 (PMC10334833; doi:10.7150/thno.80801)
Supplement: Supplementary file 1 — Supplementary figures. [file thnov13p3872s1.pdf]

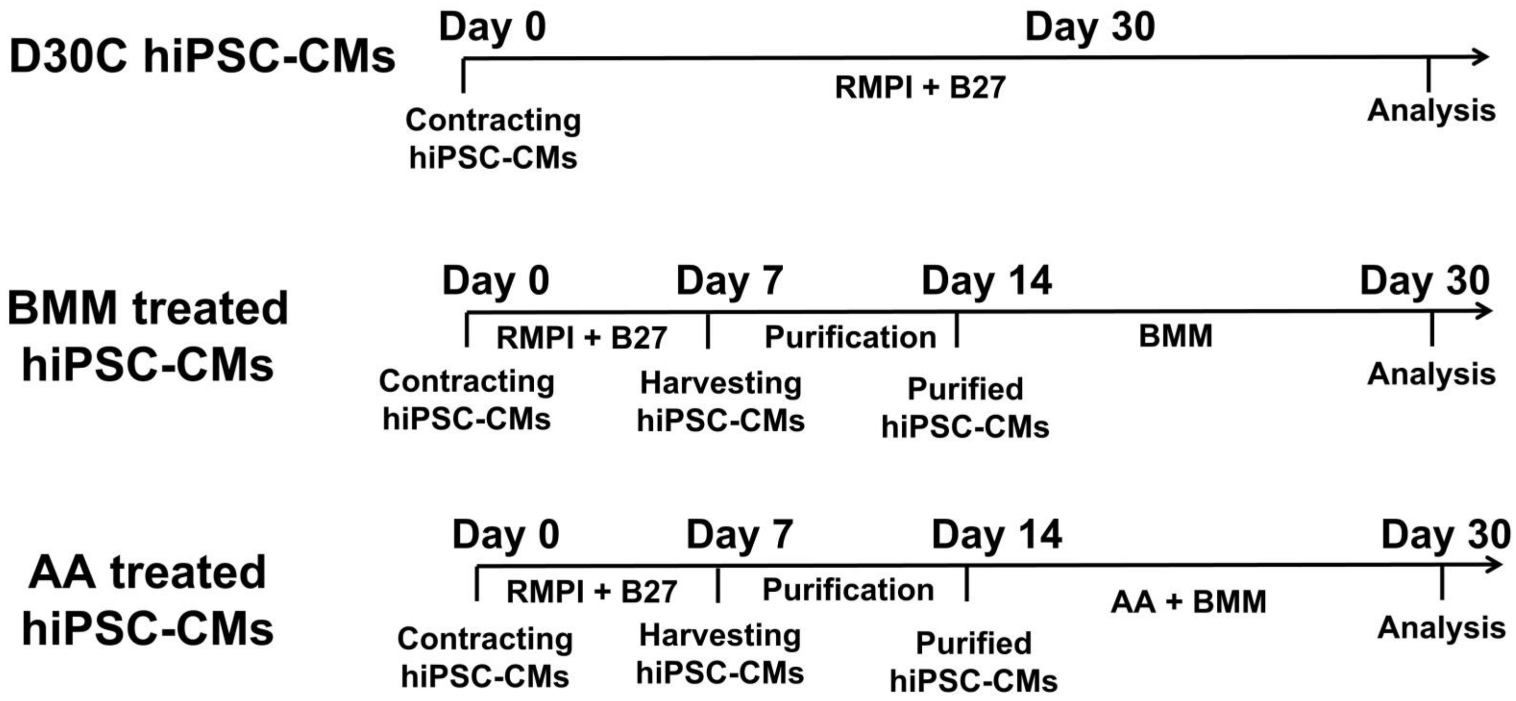

Figure S1. Schematic diagrams of the hiPSC-CM with different treatments.

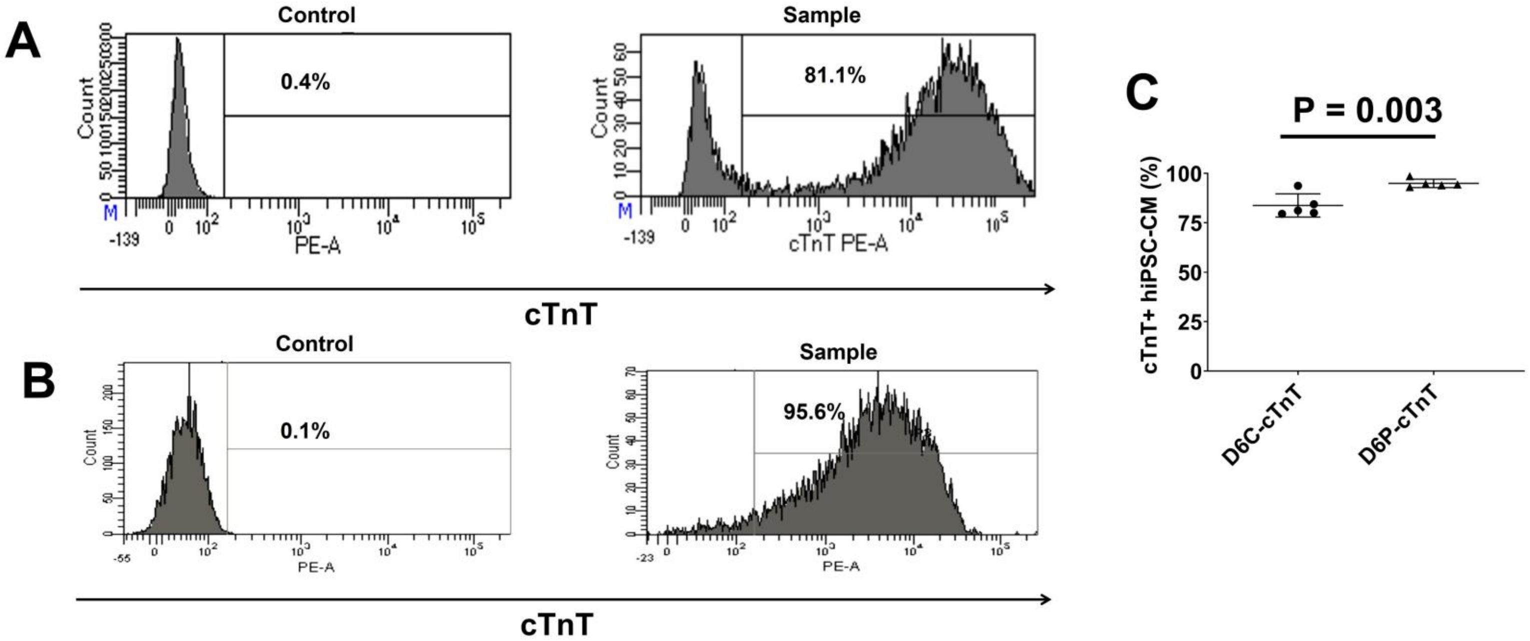

Figure S2. Purity of hiPSC-CMs. Representative flow cytometry images for assessing purity of hiPSC-CMs based on cardiac troponin T (cTnT) expression before **(A)** and after **(B)** purification. **(C)** Quantification of $cTnT^{+}$hiPSC-CM before (D6C) and after purification (D6P). (n = 5 each. Values are presented as the means ± SD. Independent T-test).

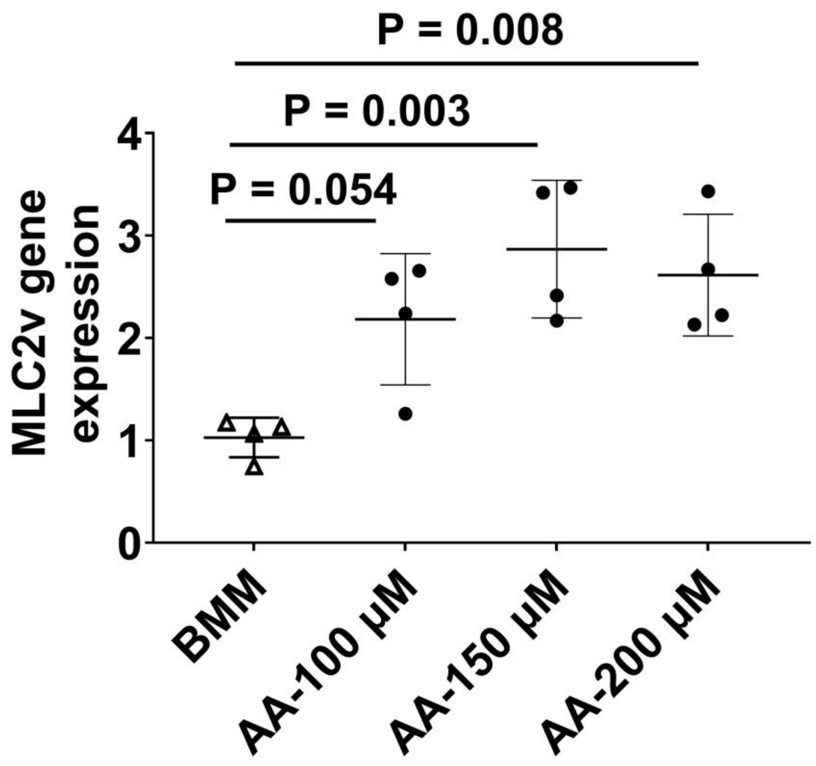

Figure S3. Dose dependent effect of ascorbic acid on MLC2v gene expression on day 7 after treatment. (n = 4 each. Values are presented as the means ± SD. One-way ANOVA)

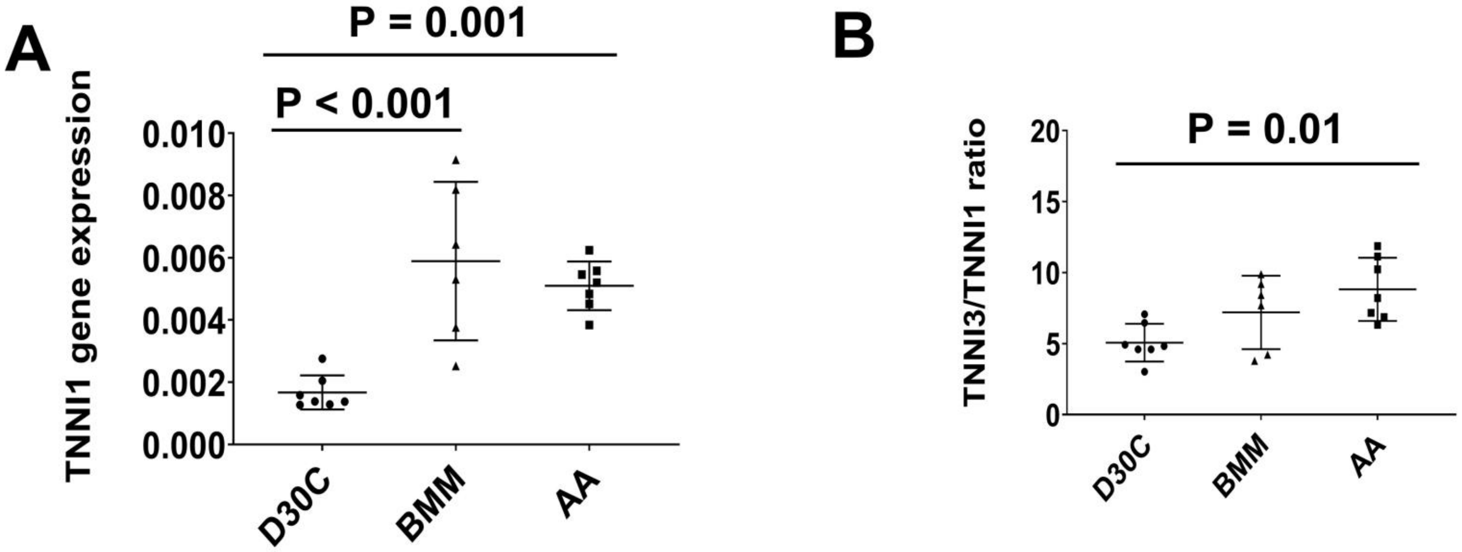

Figure S4. Gene expression levels of TNNI1 **(A)** and TNNI3/TNNI1 ratio **(B)** in D30C, BMM, or AA treated PCBC-CMs. (n = 6 or 7. Values are presented as the means ± SD. One-way ANOVA).

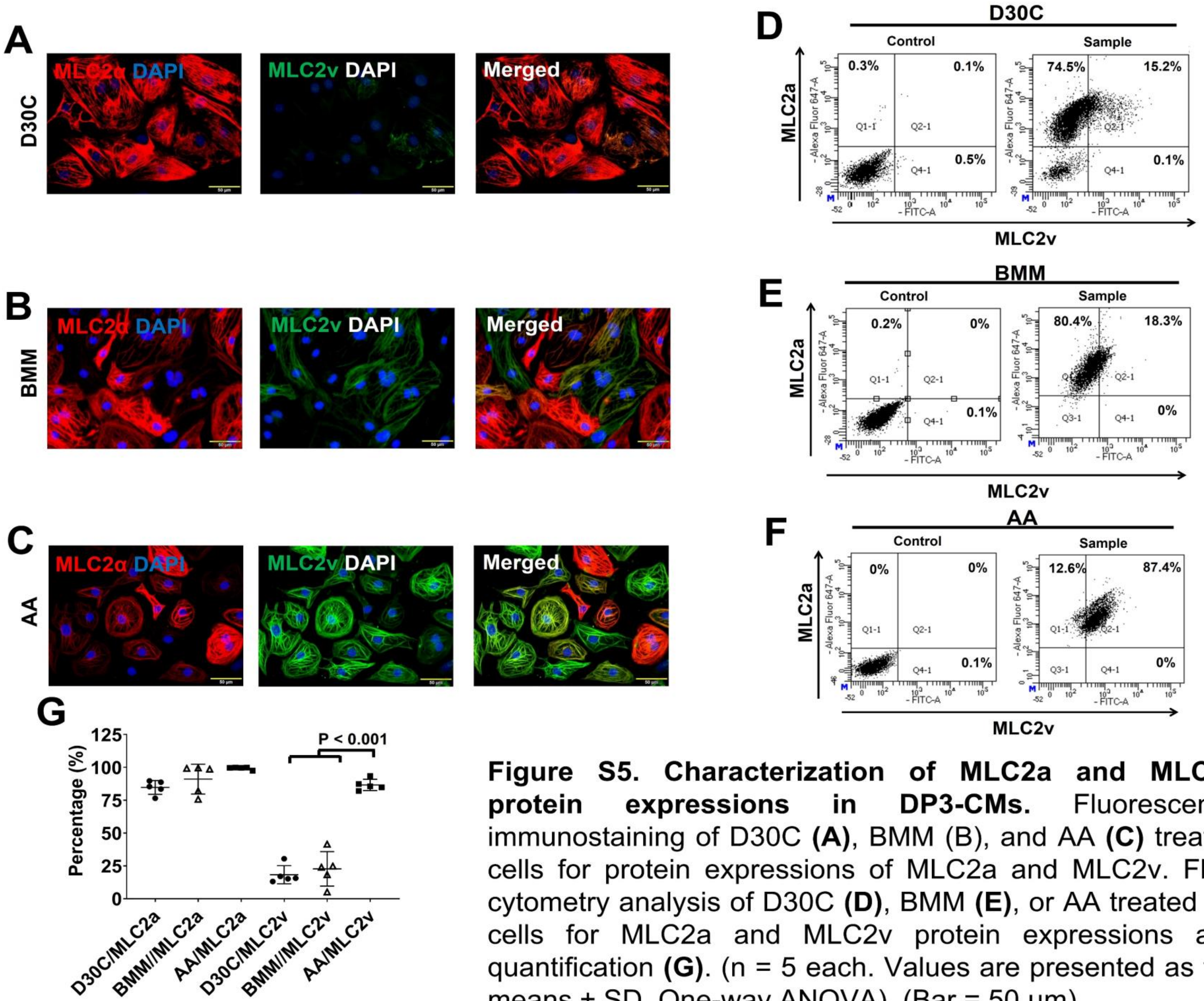

Figure S5. Characterization of MLC2a and MLC2v protein expressions in DP3-CMs. Fluorescence immunostaining of D30C **(A)**, BMM (B), and AA **(C)** treated cells for protein expressions of MLC2a and MLC2v. Flow cytometry analysis of D30C **(D)**, BMM **(E)**, or AA treated **(F)** cells for MLC2a and MLC2v protein expressions and quantification **(G)**. (n = 5 each. Values are presented as the means ± SD. One-way ANOVA). (Bar = 50 μm).

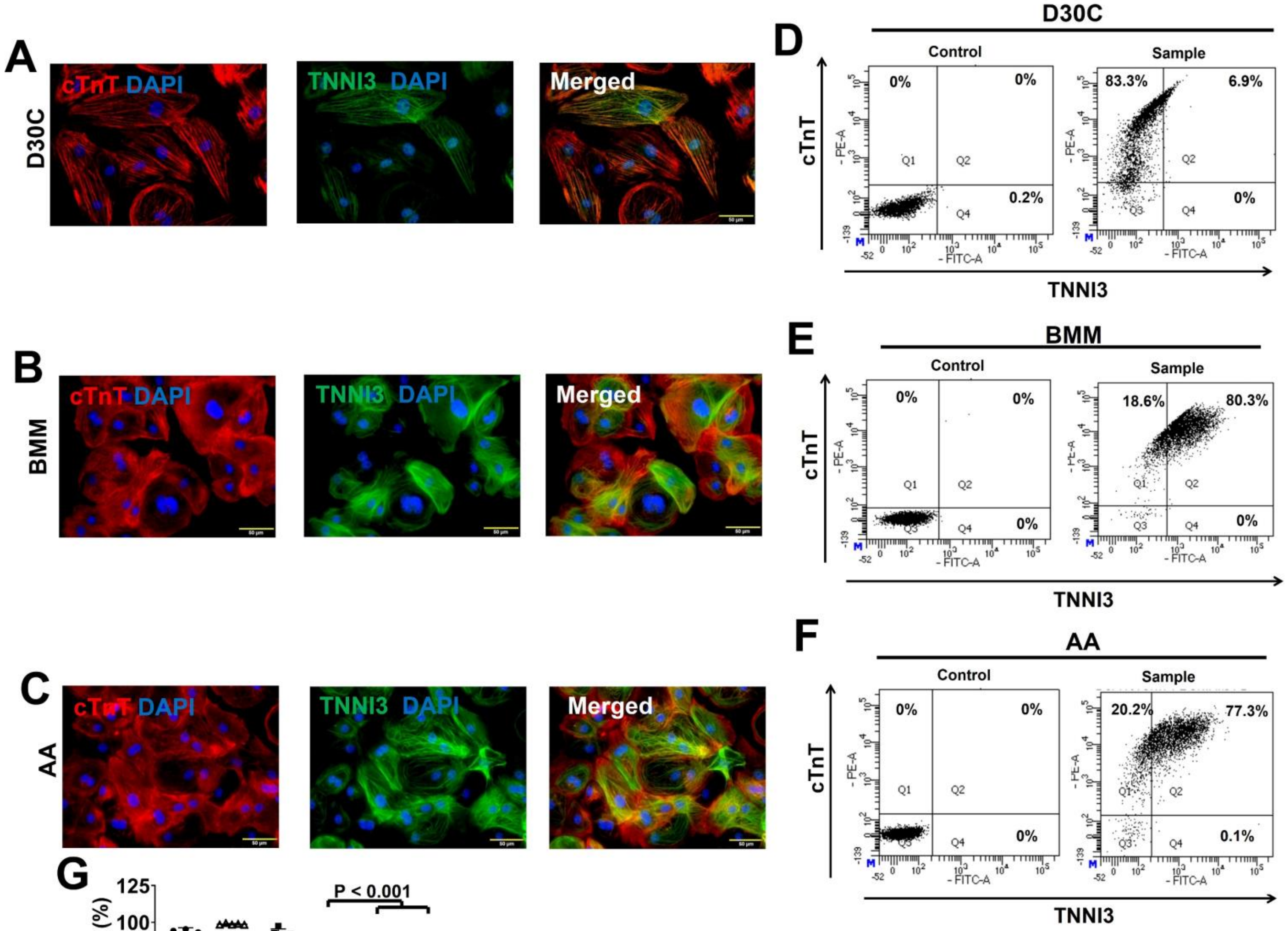

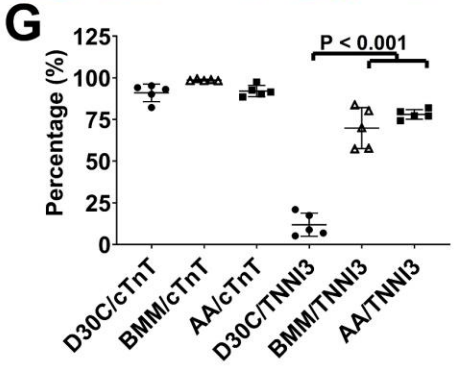

Figure S6. Characterization of cTnT and TNNI3 protein expressions in DP3-CMs. Fluorescence immunostaining of D30C **(A)**, BMM **(B)**, and AA **(C)** treated cells for protein expressions of cTnT and TNNI3. Flow cytometry analysis of D30C **(D)**, BMM **(E)**, or AA treated **(F)** cells for cTnT and TNNI3 protein expressions and quantification **(G)**. (n = 5 each. Values are presented as the means ± SD. One-way ANOVA). (Bar = 50 μm).

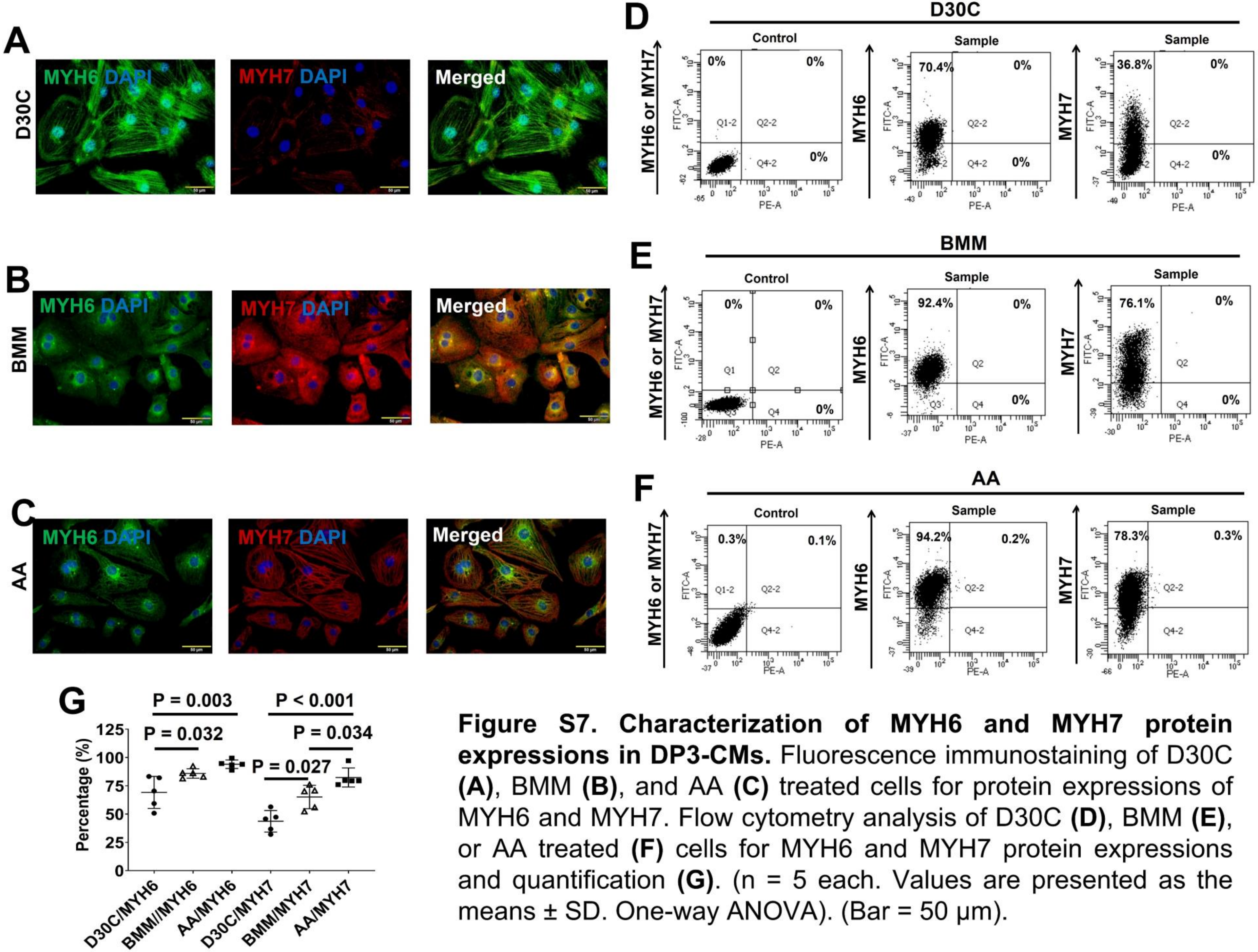

Figure S7. Characterization of MYH6 and MYH7 protein expressions in DP3-CMs. Fluorescence immunostaining of D30C **(A)**, BMM **(B)**, and AA **(C)** treated cells for protein expressions of MYH6 and MYH7. Flow cytometry analysis of D30C **(D)**, BMM **(E)**, or AA treated **(F)** cells for MYH6 and MYH7 protein expressions and quantification **(G)**. (n = 5 each. Values are presented as the means ± SD. One-way ANOVA). (Bar = 50 μm).

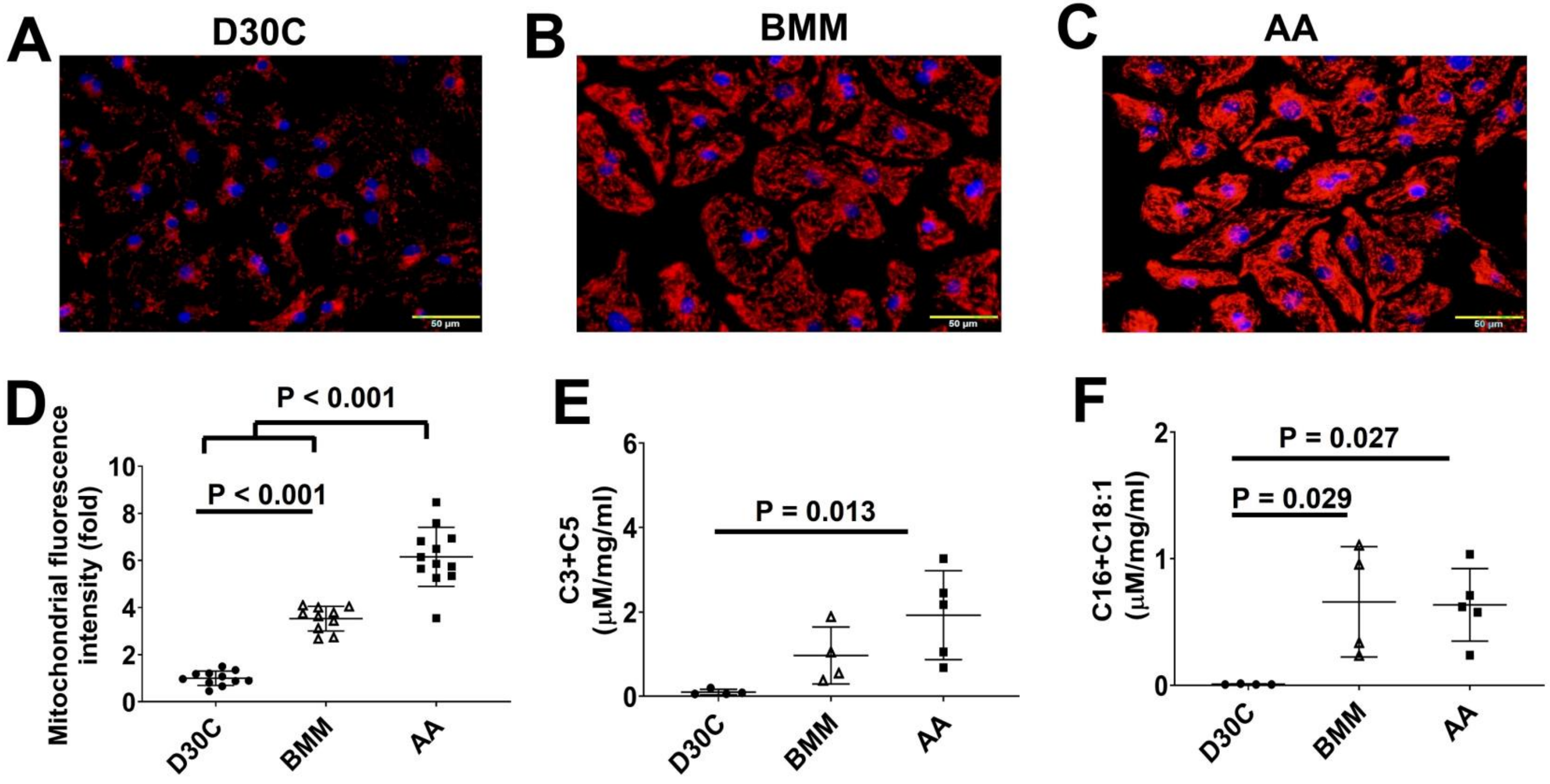

Figure S8. DP3-CM mitochondrial membrane potential and metabolism. JC-1 staining of D30C **(A),** BMM **(B),** and AA **(C)** treated DP3-CMs to visualize mitochondrial membrane potential. **(D)** Quantification of DP3-CM mitochondrial membrane potential. Metabolism of C3+C5 **(E)** and C16+C18:1 **(F)** in D30c, BMM or AA treated PCBC-CMs. (n = 10 - 12 for panel D and n = 4 - 5 for panels E & F. Values are presented as the means ± SD. One-way ANOVA). (Bar = 50 µm).

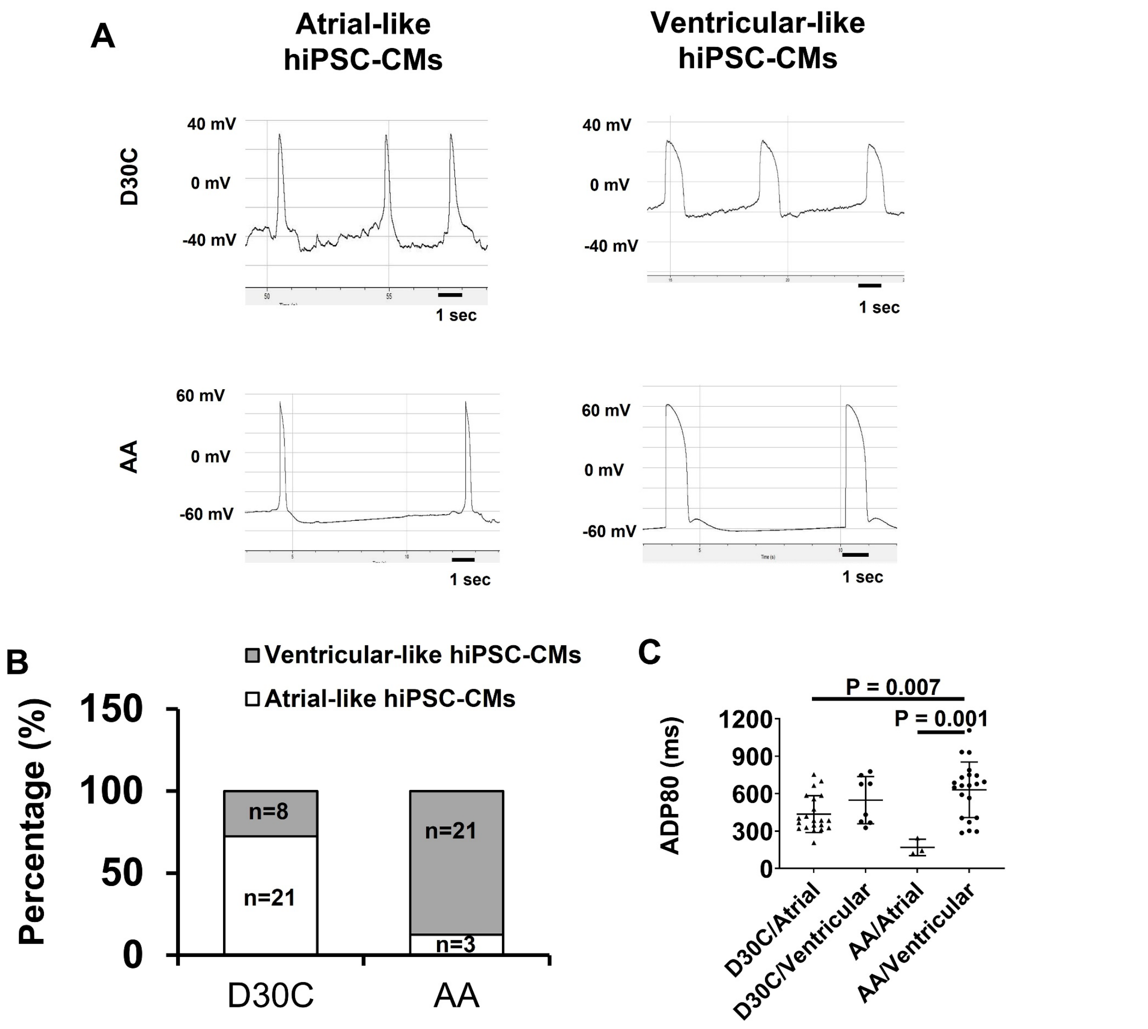

Figure S9. Patch clamp analysis of D30C and AA treated DP3-CMs. **(A)** Action potential of atrial or ventricular-like cells in D30C or AA treated DP3-CMs. **(B)** Percentage of atrial or ventricular-like cells in D30C or AA treated DP3-CMs based on action potential. **(C)** ADP80 of atrial or ventricular-like cells in D30C or AA treated DP3-CMs.

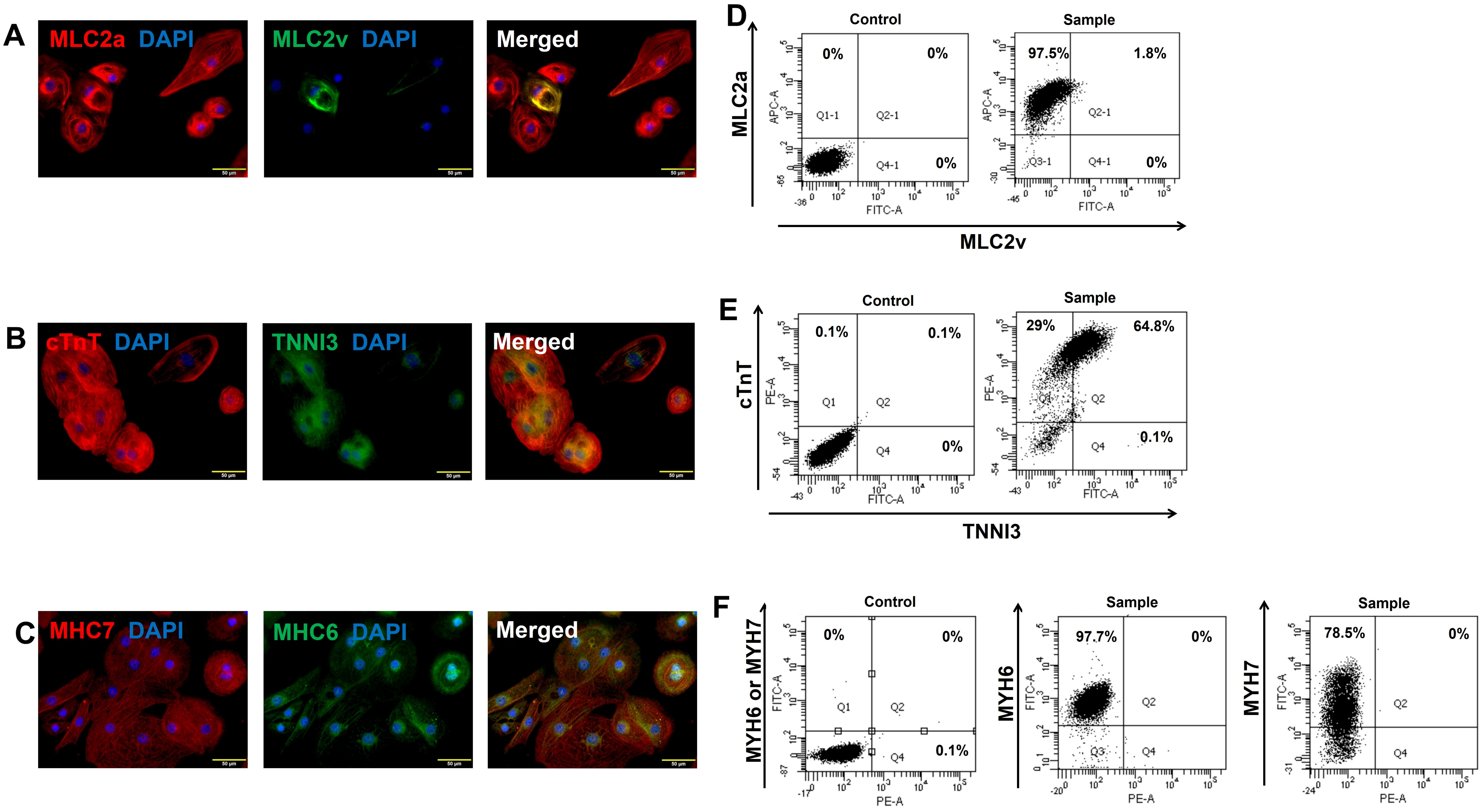

Figure S10. Fluorescence immunostaining of TETi/AA treated DP3-CMs for protein expressions of MLC2a and MLC2v **(A)**, cTnT and TNNI3 **(B)**, and MYH6 and MYH7 **(C).** Flow cytometry analysis of TETi/AA treated DP3-CMs for protein expressions of MLC2a and MLC2v **(D)**, cTnT and TNNI3 **(E)**, and MYH6 and MYH7 **(F).** (Bar = 50 μm).

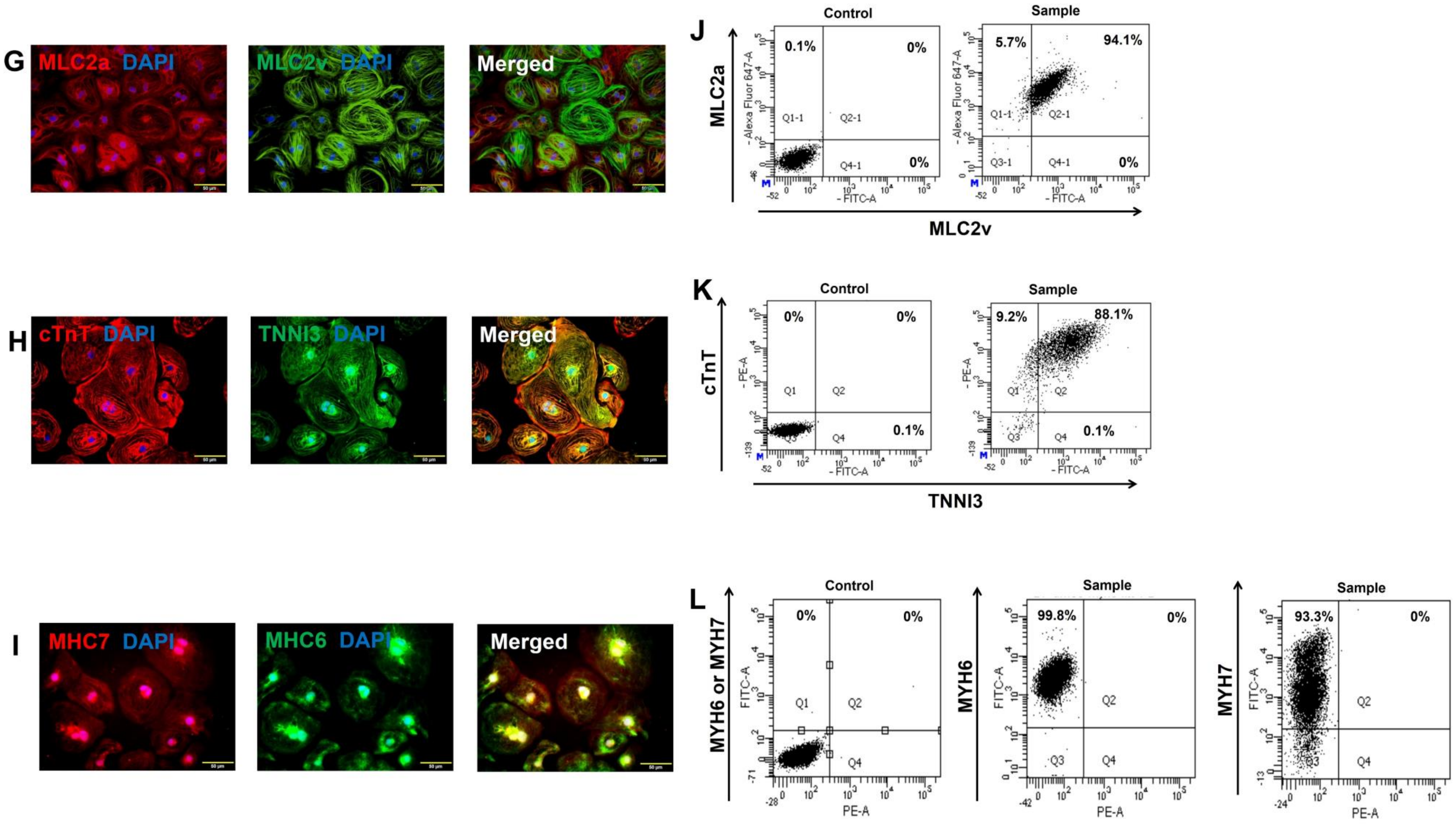

Figure S10. Fluorescence immunostaining of DMSO/AA treated DP3-CMs for protein expressions of MLC2a and MLC2v **(G)**, cTnT and TNNI3 **(H)**, and MYH6 and MYH7 **(I).** Flow cytometry analysis of DMSO/AA treated DP3-CMs for protein expressions of MLC2a and MLC2v **(J)**, cTnT and TNNI3 **(K)**, and MYH6 and MYH7 **(L).** (Bar = 50 µm).

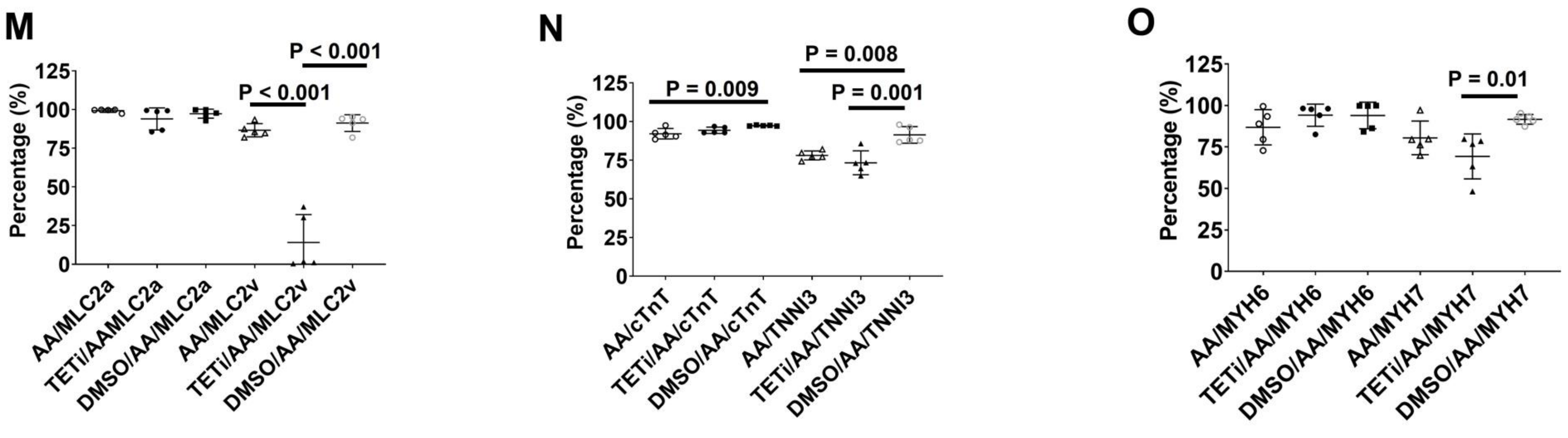

Figure S10. Quantification of AA, TETi/AA, and DMSO/AA treated DP3-CMs for protein expressions of MLC2a and MLC2v **(M),** cTnT and TNNI3 **(N)**, and MYH6 and MYH7 **(O)**. (n = 5 each. Values are presented as the means ± SD. One-way ANOVA).

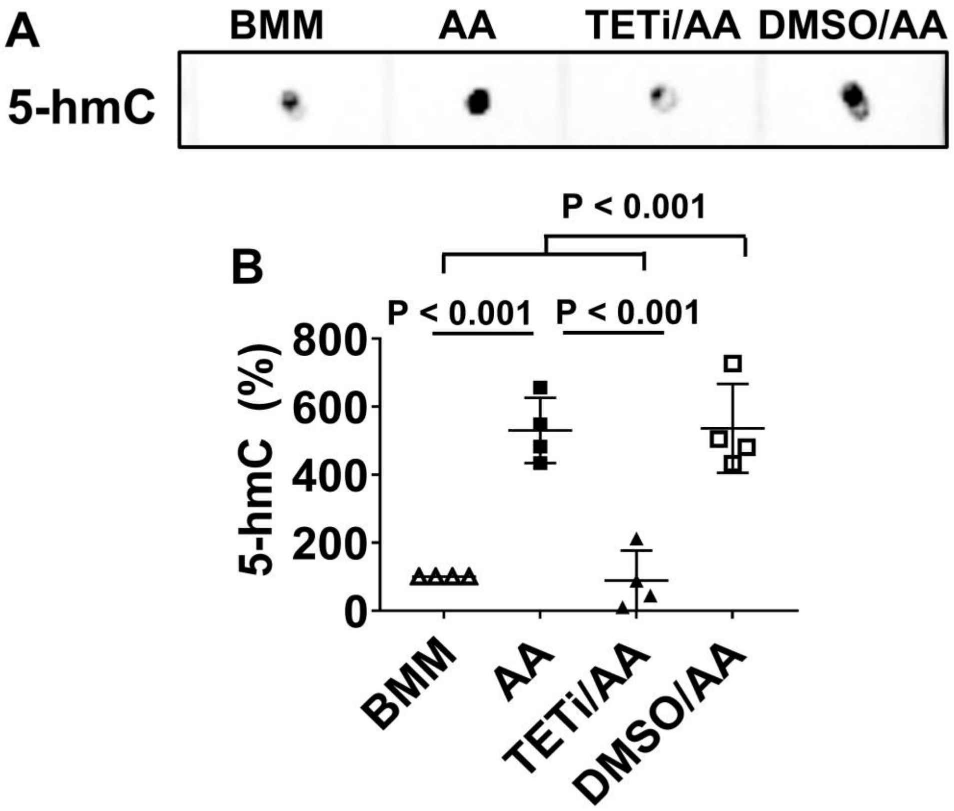

Figure S11. DNA dot plot analysis. Representative images of DNA dot-plot analysis **(A)** and quantification **(B)** for 5-hmC expression in BMM, AA, TETi/AA, or DMSO/AA treated DP3-CMs. (n = 4, values are presented as the means ± SD. One-way ANOVA).
